# Sulfur Respiration in a Group of Facultatively Anaerobic Natronoarchaea Ubiquitous in Hypersaline Soda Lakes

**DOI:** 10.3389/fmicb.2018.02359

**Published:** 2018-10-02

**Authors:** Dimitry Y. Sorokin, Enzo Messina, Violetta La Cono, Manuel Ferrer, Sergio Ciordia, Maria C. Mena, Stepan V. Toshchakov, Peter N. Golyshin, Michail M. Yakimov

**Affiliations:** ^1^Winogradsky Institute of Microbiology, Research Centre of Biotechnology, Russian Academy of Sciences, Moscow, Russia; ^2^Department of Biotechnology, Delft University of Technology, Delft, Netherlands; ^3^Institute for Coastal Marine Environment, National Research Council, Messina, Italy; ^4^Institute of Catalysis, Spanish National Research Council, Madrid, Spain; ^5^Proteomics Unit, National Center for Biotechnology, Spanish National Research Council, Madrid, Spain; ^6^Immanuel Kant Baltic Federal University, Kaliningrad, Russia; ^7^School of Biological Sciences and The Centre for Environmental Biotechnology, Bangor University, Bangor, United Kingdom

**Keywords:** polysulfide reductase, haloarchaea, hypersaline soda lakes, sulfur respiration, hydrogenase, CISM, molybdopterin oxidoreductases, facultative anaerobic

## Abstract

The ubiquity of strictly anaerobic sulfur-respiring haloarchaea in hypersaline systems with circumneutral pH has shaken a traditional concept of this group as predominantly aerobic heterotrophs. Here, we demonstrated that this functional group of haloarchaea also has its representatives in hypersaline alkaline lakes. Sediments from various hypersaline soda lakes showed high activity of sulfur reduction only partially inhibited by antibiotics. Eight pure cultures of sulfur-reducing natronoarchaea were isolated from such sediments using formate and butyrate as electron donors and sulfur as an electron acceptor. Unlike strict anaerobic haloarchaea, these novel sulfur-reducing natronoarchaea are facultative anaerobes, whose metabolic capabilities were inferred from cultivation experiments and genomic/proteomic reconstruction. While sharing many physiological traits with strict anaerobic haloarchaea, following metabolic distinctions make these new organisms be successful in both anoxic and aerobic habitats: the recruiting of heme-copper quinol oxidases as terminal electron sink in aerobic respiratory chain and the utilization of formate, hydrogen or short-chain fatty acids as electron donors during anaerobic growth with elemental sulfur. Obtained results significantly advance the emerging concept of halo(natrono)archaea as important players in the anaerobic sulfur and carbon cycling in various salt-saturated habitats.

## Introduction

Extremely halophilic archaea representing the class *Halobacteria* within the phylum *Euryarchaeota* are predominant inhabitants in salt-saturated environments. They are surprisingly diverse in their ecophysiology in relation to the carbon metabolism and particularly to the type of respiration. Most of the known *Halobacteria* species are aerobic heterotrophs (Andrei et al., 2012; Oren, 2013, 2014) with only a few species capable of sugar or arginine fermentation and anaerobic respiration with nitrate, fumarate, dimethyl sulfoxide (DMSO) or trimethylamine *N*-oxide (TMAO) as electron acceptors (Oren and Trüper, 1990; Oren, 1991; Antunes et al., 2008; Bonete et al., 2008; Werner et al., 2014). Despite molecular evidences of the presence of diverse uncultured haloarchaeal lineages in anoxic sediments (Walsh et al., 2005; Youssef et al., 2011; Lamarche-Gagnon et al., 2015), their isolation and eco-physiological studies have resisted until recently.

Our recent cultivation approach, which used sulfur compounds as electron acceptors, was successful to uncover two novel functional groups of strictly anaerobic haloarchaea. The first group, described as *Halanaeroarchaeum sulfurireducens* (HAA), uses acetate as an electron donor for elemental sulfur-dependent respiration – a catabolic route, previously overlooked in the whole archaeal Domain (Messina et al., 2016; Sorokin et al., 2016a,b). The second group, described as *Halodesulfurarchaeum formicicum* (HDA), represents the first example of lithoheterotrophy in haloarchaea. These organisms can use formate or H_2_ as the electron donor and elemental sulfur, thiosulfate or DMSO as the electron acceptor (Sorokin et al., 2017b). The discovery of these two ubiquitous groups of strictly anaerobic sulfur-respiring haloarchaea living in anoxic sediments of hypersaline lakes showed that the existing concept of the haloarchaeal physiology requires revision.

Apart from hypersaline NaCl- and Na_2_SO_4_-rich habitats with circumneutral pH values, there is also a special type of hypersaline alkaline lakes, whereby soluble sodium carbonates constitute a substantial part of the total salt in the brines reaching in some cases molar concentrations and resulting in stable, highly alkaline pH values ranging from 9 to 11 (Hammer, 1986; Grant, 2006; Sorokin et al., 2011, 2015; Schagerl and Renaut, 2016). One of the important consequences of such conditions for the anaerobic sulfur cycle is the increased chemical stability of polysulfides ($S_{x}^{-}$), which obviates limitations of utilization of elemental sulfur as electron acceptor at circumneutral pH. We have previously shown that microbial sulfur/polysulfide reduction in the sediments of soda lakes is the most active process in the anaerobic sulfur cycle. Notably, in some soda lake sediment incubation the maximum rates of sulfur reduction were observed when formate was added as the electron donor and when salinity was approaching saturation (Sorokin et al., 2010, 2012). This hinted at the involvement of extreme natronophiles in formate-dependent sulfidogenesis and was a starting point for the discovery of sulfur-reducing natronoarchaea reported in this study. To have a first glimpse into the metabolic capabilities of this physiologically novel haloarchaeal ecotype, we performed physiological characterization and genome assemblage of three isolates and assessed their key respiratory properties via genome-wide proteomic studies of cultures grown on different couples of electron acceptors and donors.

## Materials and Methods

### Samples and Sediment Activity Incubation

Top 10 cm sediment and brine samples were obtained from several hypersaline soda lakes in the *NE* Mongolia and the Kulunda Steppe (Altai region, *SW* Siberia, Russia), from hypersaline alkaline lakes in Egypt (Wadi al Natrun) and California (Searles and Owens Lakes). The main chemical characteristics of these lakes are given in the **Supplementary Table S1**. Sediment samples from the Kulunda Steppe soda lakes were collected into a 20 cm plastic corer with a diameter of 6 cm and extruded into a sterile 200 ml Schott flask, closed without bubbles and transported into the laboratory in insulated box with cooling elements within 3 days after sampling. Samples from other lakes were collected with a 50 ml plastic syringe with cut end into 50 ml sterile Falcon tubes and closed avoiding air bubbles. After arrival to the laboratory, the samples were transferred immediately into glass bottles closed with rubber stoppers and were kept under argon at 12°C until processing.

To assess potential sulfur-reducing activity, 1:1 (vol/vol) slurries were prepared from surface sediments (5–10 cm deep) and near bottom brines (1:1, vol/vol) from two soda lakes in Kulunda Steppe (Altai, Russia) taken in 2011: a moderately saline Cock soda lake and a hypersaline Bitter-1 lake. 10 ml slurries were dispensed into 23 ml serum bottles and 10 mg powdered sulfur and various e-donors at 2–5 mM were added. The bottles, closed with butyl rubber stoppers, were made anoxic by 3 cycles of evacuation-flushing with argon gas and incubated statically with periodic hand mixing at 25°C for 3–7 days with regular monitoring of sulfane (a sum of free HS^-^ and sulfane of polysulfides) formation. Same setup was used to investigate the influence of salinity on the sulfur reduction by replacing the native pore brines of the sediments with a sodium carbonate buffer at pH 10 containing a range of total Na^+^ from 0.2 to 4.5 M and using formate as the *e*-donor.

### Enrichment and Cultivation Conditions

Two mineral basic media (4 M total Na^+^) were used in different proportions to achieve variable pH and sodium carbonate : NaCl ratio. The NaCl base medium, pH 7, contained (g L^-1^) : NaCl – 240; KCl – 5, K_2_HPO_4_ – 2; NH_4_Cl – 0.5. The sodium carbonate basic medium, pH 10, contained (g L^-1^): Na_2_CO_3_ – 190, NaHCO_3_ – 30, NaCl – 16, KCl – 5.0 and K_2_HPO_4_ – 1.0. After sterilization both basic media were supplemented with 1 mM MgCl_2_ x 6H_2_O, 1 ml L^-1^ of acidic trace metal solution and vitamin mix (Pfennig and Lippert, 1966), 1 mL L^-1^ of alkaline Se/W solution (Plugge, 2005) and 20 mg L^-1^ of yeast extract. 4 mM NH_4_Cl was also added to the sodium carbonate basic medium. The two basic media were mixed 1: 1 (pH final ∼9.6) for soda lake enrichments and isolates and at 3 NaCl: 1 Na_2_CO_3_ bases (final pH 9.3) for the Searles Lake enrichment. The pH range for growth and activity at 4 M total Na^+^ was created by adding 50 mM HEPES to the NaCl base (pH from 6 to 8), combining two basic media in different proportions (pH 8.5–9.5) and by titrating 1:1 mix of two bases from pH 9.5 up to 11 by adding 4M NaOH. The salt profiling was checked at optimal pH by using 5M NaCl basic medium instead of 4M to exceed the 4M Na^+^ concentrations. Elemental sulfur flour was sterilized at 110°C for 30 min as a wet paste and added to approximately 2 g L^-1^. 1M DMSO stock solution was filter-sterilized and added at 10 mM (final concentration). Other tested *e*-donors/acceptors were added from sterile anaerobic 1M stock solutions by syringe at final concentrations 5–10 mM. Formate was supplied at final concentration 50 mM. Cultivation was performed at 37°C in 12–120 ml serum bottles with butyl rubber stoppers filled with liquid to 90% in case of formate and 50% in case of H_2_. The sterile medium was made anoxic first by “cold boiling” upon evacuation followed by 3 cycles of flushing with sterile argon and evacuation. Anaerobic conditions were achieved by final addition of 0.2 mM HS^-^. H_2_ was added through sterile gas filter at 0.5 bar overpressure on the top of argon atmosphere. The cultures were incubated at 37°C with periodic shaking of the flasks. The ability for aerobic growth was tested in the absence of sulfur and sulfide, and with liquid to gas ratio of 1:10. 0.2 g L^-1^ yeast extract and peptone each were added in addition to carbon and energy growth substrates. The final concentration of O_2_ in the gas phase varied from 1 to 20%. Pure cultures were obtained by serial dilutions to extinction and the final isolates were checked microscopically and by 16S rRNA sequencing. Colony formation was possible only on Petri plates incubated in anaerobic jars at microaerobic conditions (2%). Natronoarchaeal strain 93dLM4, isolated as a proteolytic aerobe from Little Lake Magadi in Kenya (Duckworth et al., 1996), was kindly provided by B. E. Jones. Aerobic cultures of *Natronobacterium gregoryi*, *Natronolimnobius baerhaense*, and *Nl. innermongolicus* were obtained from the Deutsche Sammlung von Mikroorganismen und Zellkulturen (DSMZ) and Japan Collection of Microorganisms (JCM).

### Chemical and Microscopic Analyses

Sulfide/sulfane formation was measured by using methylene blue method (Trüper and Schlegel, 1964) after fixing 0.1 mL slurry or culture supernatant in 1 mL 10% Zn acetate. Internal sulfur in polysulfide was recovered after centrifugation of acidified supernatant to pH 3.0 and extraction of the formed sulfur pellet with acetone overnight followed by colorimetric determination as FeSCN complex (Sörbo, 1957). Formate consumption and acetate formation from butyrate in sulfur-reducing cultures were analyzed by HPLC (BioRad HPX-87H column at 60°C; eluent 1.5 mM H_3_PO_4_, 0.6 ml min^-1^; UV/RI detector Waters 2489) after cell removal, acidification of the supernatant to pH 5 to destroy polysulfides and filtration through HPLC filters. The final preparation had to be diluted 5–10 times because of very high salinity. The cell protein was determined by the Lowry method in 1–2 mL culture samples after centrifugation 13,000 rpm for 20 min. The cell pellets were washed with 4 M NaCl at pH 5 to remove the cell-bound FeS. Microbial growth was followed by increase in optical density at 600 nm (OD_600_). Cytochrome absorption spectra were recorded on the UV-Visible diode-array HP 8453 spectrophotometer (Hewlett Packard, Amsterdam, Netherlands).

Phase contrast microphotographs were obtained with a Zeiss Axioplan Imaging 2 microscope (Göttingen, Germany). Intracellular polyhydroxyalkanoate inclusions were identified by Nile-Blue staining (Johnson et al., 2009). For total electron microscopy, the washed cells were fixed with paraformaldehyde and stained with 1% uranyl acetate. For thin sectioning, the cells were fixed in 1% (w/v) OsO_4_ containing 3.0 M NaCl for 96 h at 8°C, washed with 4 M NaCl, stained overnight with 1% (w/v) uranyl acetate in 3 M NaCl, dehydrated in an increasing ethanol series, and embedded in Epon resin. Thin sections were stained with 1% (w/v) lead citrate.

### Genome Sequencing, Assembly and Annotations

The complete genomes of *N. sulfurireducen*s AArc1, AArc-Mg, and *H. desulfuricum* AArc-Sl were sequenced with the MiSeq System of Illumina Inc. (San Diego, CA, United States) using a combination of both short insert (2 × 250 bp) and 3–5 kbp-long insert (mate pair, 2 × 150 bp) paired-end reads (MiSeq Reagent Kit v2). The obtained reads, equivalent to genome coverage of about 129x for AArc1, 112x for AArc-Mg, and 66x for AArc-Sl, were respectively assembled by SPAdes 3.7.1 (Bankevich et al., 2012) and Velvet 1.2.10 (Zerbino and Birney, 2008) programs, and refined with contigs assembled by Geneiuos 7.1 software (Biomatters Ltd, New Zealand). Protein genes, rRNA operons, tRNAs and CRISPR regions were respectively predicted by Glimmer 3.02 (Delcher et al., 2007), RNAmmer 1.2 online server (Lagesen et al., 2007), tRNAscan-SE 2.0 online tool (Lowe and Chan, 2016), and CRISPRfinder online program (Grissa et al., 2007). Further checks for annotation consistency were performed using FgenesB online tool for operon prediction (Solovyev and Salamov, 2011), PATRIC/RAST server (Aziz et al., 2008) and NCBI blastx against KEGG database (Altschul et al., 1997)^1^ for overall annotation, and Artemis 16.0 software (Rutherford et al., 2000) for final manual check. Maximum Likelihood tree of 42 haloarchaeal genomes was constructed in the same manner described in Sorokin et al. (2015), by selecting six ribosomal conserved genes concatenated to form a sequence ready to be aligned with Clustal W 2.1 (Larkin et al., 2007) and then constructed using PhyML 3.0 (Guindon et al., 2010) with Jukes-Cantor substitution model and 1000 bootstrap resampling. 16S rRNA gene phylogeny of the AArc strains was computed from a 16S rRNA gene sequence alignment with PAUP^∗^4.b10 using a LogDet/paralinear distance method as it described elsewhere (Sorokin et al., 2016a). Maximum Likelihood phylogenetic trees of CISM catalytic subunits A and full-length HydB subunit of H_2_-uptake [NiFe]-hydrogenases were constructed with MacVector 11.1.2 and as above. The bootstrap procedure with 1000 replicates was used.

### Proteomic Analyses

Shotgun proteomic analyses were conducted using strain AArc1 grown in duplicates under following conditions: aerobically (100 mL medium in 500 mL bottle closed with a rubber septa, shaking speed 130 rpm) with 10 mM butyrate and 0.2 g L^-1^ of yeast extract; anaerobically with 50 mM formate as *e*-donor, 100 mg L^-1^ yeast extract as carbon source and either sulfur (2 g L^-1^) or 20 mM DMSO as the *e*-acceptors. The incubation temperature was 37°C and the biomass was harvested by centrifugation (14,300 × *g*, 20 min, 4°C) at 50–70% of donor lost and processed separately for shotgun proteomic analysis. Details on the methodological procedures used (e.g., protein extraction, protein concentration, in-gel trypsin digestion and nano-liquid chromatography tandem mass spectrometry) are given in previous studies (Sorokin et al., 2017a,b; Ferrer et al., 2018). The exponentially modified Protein Abundance Index (emPAI) was used as a relative quantitation score of the proteins (Ferrer et al., 2018). For comparative proteomics, normalized (nemPAI) values were obtained from the emPAI values by dividing each individual value by the sum of all emPAI values in a given experiment (Arike and Peil, 2014). To make the scale commensurate with the average protein abundance, obtained values were then multiplied by the number of proteins in AArc1, distinct by their peptide composition in all three experimental settings (Ferrer et al., 2018).

### Data and Strains Deposition

16S rRNA gene sequences were deposited on NCBI GenBank database (accession no. from KY612364 to KY612370). The complete genomes of three sulfur-reducing natronoarchaeal strains were submitted in GenBank by the NCBI Genome submission portal. Strain AArc1 has accession numbers CP024047 for chromosome, CP024045 for plasmid pAArc1-01, and CP024046 for plasmid pAArc1-02. Strain AArc-Mg has accession numbers CP027033 for chromosome and CP027032 for plasmid pAArc-Mg-01. Strain AArc-Sl has the accession number CP025066. The AArc isolates have been deposited in the UNIQEM culture collection (Collection of Unique Extremophilic Microorganisms, Russian Academy of Sciences, Moscow, Russia). The type strains AArc1 (UNIQEM U932^T^) and AArc-Sl (UNIQEM U999^T^) were also deposited in Japan Collection of Microorganisms under the numbers JCM 30663^T^ and JCM 30664^T^, respectively.

## Results and Discussion

### Potential Sulfur Reduction in Sediment Slurries

**FIGURE 1:**
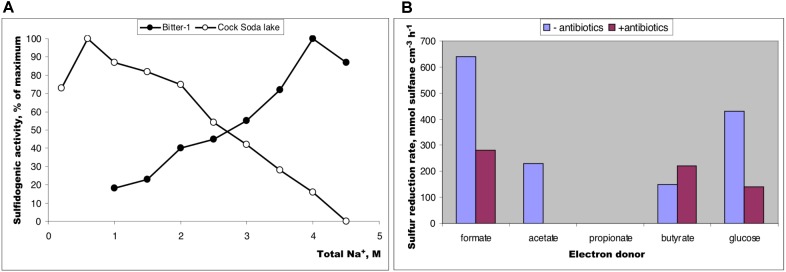
Sulfur-reducing activity in anaerobic sediment slurry mix (1:1 with brines) from soda lakes in Kulunda Steppe (2011) incubated at 25° for 3–7 days. **(A)** Influence on salinity (as sodium carbonates) on potential sulfur reduction with formate as e-donor in sediments from moderately saline Cock Soda Lake and hypersaline Bitter-1 lake; **(B)** influence of antibiotics (streptomycin + kanamycin + vancomycin, 200 mg L^-1^ each) on potential sulfur reduction is sediment slurry from a mix sample prepared from 3 hypersaline soda lakes: Tanatar-1, Bitter-1, and trona crystallizer (Kulunda Steppe, Altai, Russia). The lakes are located in a close proximity and had have a similar brine characteristics (see **Supplementary Table S1**). The maximum activity was estimated as an average value from two parallel replicated samples. Total sulfane is a sum of free HS^-^ and the sulfane atoms of polysulfides.

The salinity profiling of the potential formate-dependent sulfur-reducing activity in sediments from the two different soda lakes showed a profound difference: while the activity dropped sharply after salinity increase above 2 M total Na^+^ in the moderately saline Cock Soda Lake, it reached maximum at saturated sodium carbonate concentrations in the hypersaline Bitter-1 lake (**Figure 1A**) indicating a possible involvement of the natronoarchaea in this process in the latter lake. This was confirmed in further experiments with different *e*-donors at native hypersaline conditions using incubations with and without antibiotics. The activities with formate and glucose were only partially inhibited, while the butyrate-dependent sulfur reduction was totally insensitive to antibiotics at high concentrations (**Figure 1B**).

### Enrichment and Isolation of Pure Cultures of Sulfur-Reducing Natronoarchaea

Initial sulfur-reducing enrichments were established with sediment/brine samples from Kulunda Steppe hypersaline soda lakes at pH 9.1–11.0 and 240–400 g L^-1^ of total salinity (**Supplementary Table S1**). Formate, butyrate and peptone were chosen as electron donors. Acetate and propionate did not stimulate sulfur reduction, while the remaining substrates induced polysulfide formation within 1–4 weeks of incubation. Application of antibiotics favored elimination of the bacterial population in formate- and peptone-supplemented enrichments (*Natroniella sulfidigena* and a member of *Halanaerobiales*, respectively, as revealed by 16S rDNA monitoring), while the butyrate cultures were dominated by natronoarchaea already in primary enrichments. Further serial dilutions to extinction eventually produced three pure cultures of sulfur-reducing natronoarchaea from Kulunda Steppe soda lakes: AArc1, AHT32 and AArc-P (**Table 1**). Using the same approach, four pure cultures of sulfur-reducing natronoarchaea were obtained from similar environments that were sampled in Egypt, Mongolia, and United States. Single active sulfidogenic enrichment was obtained with brine/sediments from Searles Lake after a twofold decrease in the sodium carbonate content in the basic medium in favor of NaCl. This correlated with the much lower alkalinity of Searles Lake compared to other soda lakes sampled. Finally, a pure culture, strain AArc-Sl, was obtained using serial dilutions to extinction approach, which finalized the total number of isolates to eight (**Table 1**).

**Table 1:** Natronarchaeal sulfur-reducing strains isolated from hypersaline alkaline lakes.

Strain	Lake	*e*-donor in anaerobic enrichment	Affiliation	Deposition in culture collections
AHT32	Soda lakes (sediment + brine mix)	Butyrate	‘Natronolimnobius sulfurreducens’	U931 / JCM 17580
AArc1^T^	Trona crystallizer	Formate		U932 / JCM 30663
AArc-P	Bitter-1	Peptone		
AArc-Mg	Lakes Hotontyn / Shar-Burdiin	Butyrate		
AArc-Bj	Lake Badain	Formate		
AArc-Wn	Wadi Natrun (sediment + brine mix)	Butyrate		
AArc-Ow	Owens Lake (sediment + brine)	Butyrate		
93dlM4	Little Lake Magadi (isolated by Brian Jones)	–		
AArc-Sl^T^	Searles Lake (sediment)	Formate	‘Halalkaliarchaeum desulfuricum’	U999 / JCM 30664


U, UNIQEM culture collection (Moscow); JCM, Japan Collection of Microorganisms.

### Identification of the AArc Isolates

**FIGURE 2:**
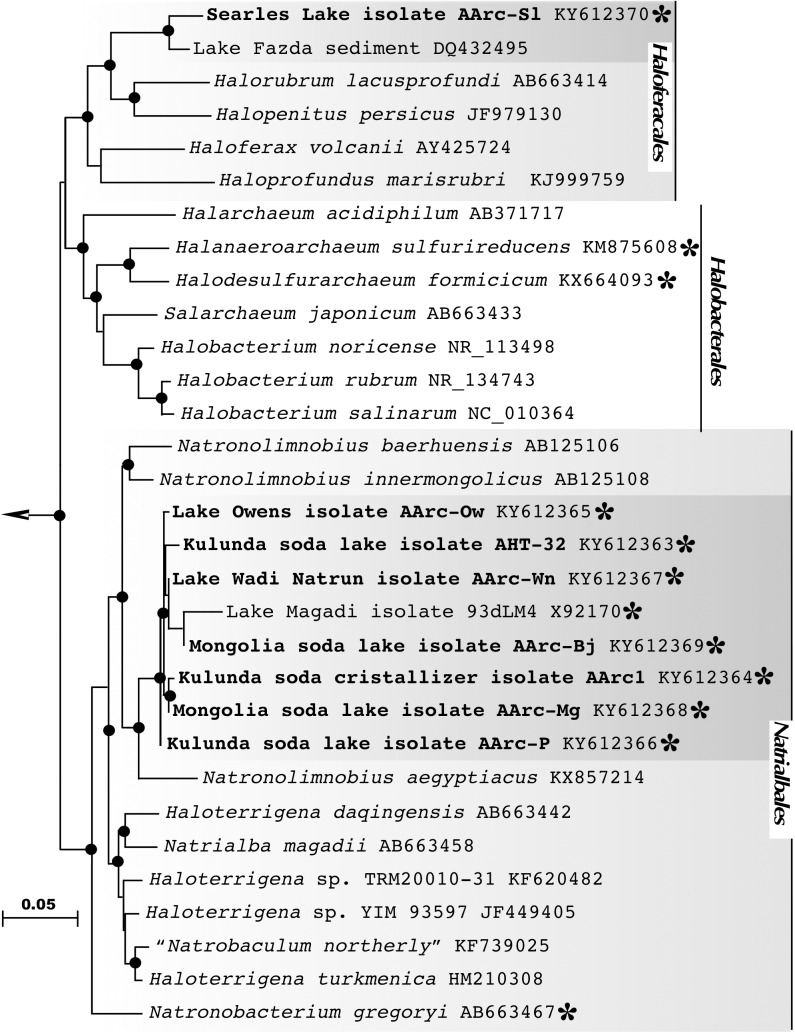
16S rRNA gene-based Maximum Likelihood phylogenetic tree showing the position of the sulfur-reducing natronoarchaea isolates (highlighted in bold) within the orders *Haloferacales* and *Natrialbales*. Members of the class *Halobacteria*, with experimentally confirmed ability for sulfur respiration, are highlighted by asterisks. Filled circles at the nodes indicate the bootstrap values > 70% (1000 bootstrap iterations). *Methanohalophilus halophilus* DSM 3094^T^ (GenBank accession no. FN870068) was used as an outgroup. Bar represents 0.05 substitutions per nucleotide position.

Analysis of the 16S rRNA gene sequences revealed that the sulfur-reducing natronoarchaeal isolates were affiliated with two distinct taxonomic clusters. The dominant cluster included seven closely related strains (98–100% 16S rRNA gene identities), which were all obtained from hypersaline soda lakes. Together with an unclassified isolate 93dLM4 from the Little Lake Magadi (Kenya), the cluster formed a distinct monophyletic lineage in a robust clade consisting of members of the genera *Natronolimnobius* (Itoh et al., 2005) in the order *Natrialbales* (**Figure 2**). The isolate AArc1^T^, chosen as a representative member of the cluster, exhibited 94.6-95.9 % 16S rRNA gene sequence identities with the three validly described species of this genus (*N. baerhuensis*, *N. innermongolicus*, and *N. aegyptiacus*), apparently representing a new species (Stackebrandt and Ebers, 2006; Kim et al., 2014; Yarza et al., 2014), for which we propose a provisional name “Natronolimnobius sulfurireducens.” Strain AArc-Sl, isolated from alkaline hypersaline Lake Searles, was forming a tight cluster with an uncultured haloarchaeal clone WN-FSA-155 (97.85% of sequence identity) from the alkaline hypersaline Lake Fazda in Wadi Natrun, Egypt (Mesbah et al., 2007). Both sequences were only distantly related to the closest genera *Halorubrum* and *Halopenitus* (<92% of sequence identity) and likely formed a novel genus-level branch within the order *Haloferacales* for which we propose a tentative name “Halalkaliarchaeum desulfuricum.”

### General Phenotypic Characteristics

The cell morphology of the isolates varied from flattened irregular coccoids to board-like rods, which were occasionally motile, depending upon the growth conditions. The cells grown with butyrate accumulated a large amount of polyhydroxyalkanoate (PHA) granules (staining positive with Nile Blue), while the cells grown with pyruvate, formate or peptone were mostly free of inclusions. (**Supplementary Figure S1**). The cells of all isolates lysed when the salt concentration was lowered below 1.5–2.0 M Na^+^.

All obtained isolates of sulfur-reducing natronoarchaea, especially AArc-Sl, had several key metabolic properties in common with previously described *Halodesulfuriarchaeum formicicum* (HDA) strains (Sorokin et al., 2017b). They were able to grow anaerobically as lithoheterotrophs using either formate or H_2_ as *e*-donors and either acetate or yeast extract (preferred) as carbon sources, while either elemental sulfur/polysulfide or DMSO (except for strain AArc-Sl) served as the terminal *e*-acceptor. However, in sharp contrast to the group of strict anaerobic sulfur-reducing haloarchaea from salt lakes (Sorokin et al., 2016a, 2017a), the natronoarchaea were facultative aerobes, albeit, the transition to aerobic conditions from sulfur-respiring cultures required gradual adaptation through several steps of static microoxic incubation with gradual increase of oxygen in the gas phase from 2 to 20%. The aerobically grown cells had intensive red-orange color in contrast to the sulfur-reducing cultures, whose biomass was beige or black (**Supplementary Figure S2**). The aerobically cultivated AArc1 cells also produced a rhodopsin. Neither formate nor H_2_ were utilized as electron donors aerobically indicating that both their H_2_-uptake hydrogenase and formate dehydrogenase belonged to anaerobic types. In contrast, the respiration with acetate was possible only during aerobic growth, while under sulfur-reducing conditions acetate was utilized exclusively as the carbon source. Compared to strict anaerobic haloarchaea, the soda lake AArc isolates were very versatile (except for strain AArc-Sl) and capable of using various electron donors for anaerobic growth with sulfur. Apart from formate, H_2_ and butyrate, they were able to take up short (C_5_-C_9_) chain fatty acids (SCFA), pyruvate, lactate, glycerol, yeast extract and peptone. Strain AArc-Sl only used H_2_/formate as donor and pyruvate as the carbon source, similarly to its haloarchaeal counterpart, *Halodesulfuriarchaeum formicicum* HSR6^T^. Testing of other electron acceptors (thiosulfate, sulfite, nitrate, nitrite, arsenate, selenate, selenite, ferrihydrite, MnO_2_) with formate as the *e*-donor produced negative results for all isolates.

**FIGURE 3:**
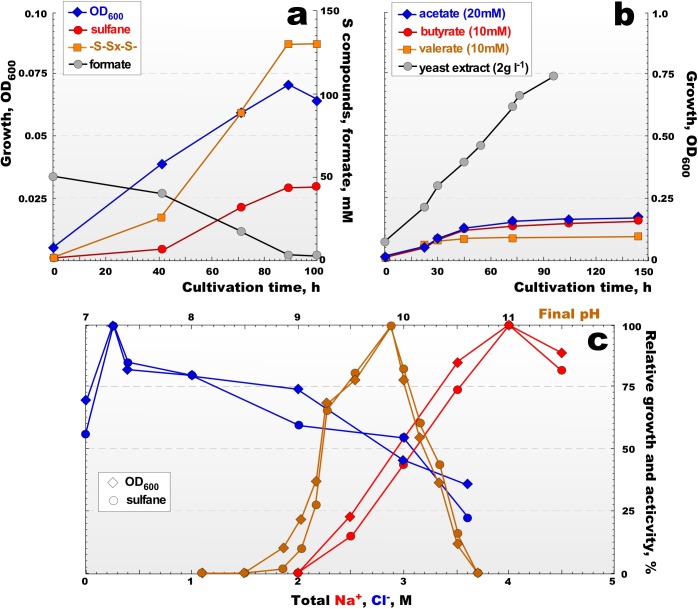
Growth kinetics and sulfidogenic activity of strain AArc1 at 4.0 M Na^+^, pH 9.5 and 37°C. **(a)** Anaerobic growth with formate as *e*-donor, elemental sulfur as *e*-acceptor and acetate 1 mM as carbon source. **(b)** Aerobic growth on acetate, butyrate, valerate and yeast extract. **(c)** Influence of pH at 4 M total Na^+^ (orange lines), of salinity at pH 9.5 (as total Na^+^, red lines) and of Cl^-^ at 4 M total Na^+^ and at pH 9.5 (blue lines) on anaerobic growth with formate + sulfur. The data are mean values from 2 replicate cultures.

All AArc strains from the main group 1 had a very similar growth profiles and characteristics while using formate as *e*-donor and sulfur as acceptor. More detailed growth physiology, therefore, was examined using two type strains - AArc1 for group 1 and AArc-Sl for group 2. The anaerobic growth dynamics of the AArc group 1 isolates was very different from those of formate-utilizing neutrophilic HDA strains. They grew faster and converted much more sulfur into the soluble form as polysulfides (average formula S_5.5_^2-^) accumulated in the medium at concentrations of up to 100 mM total sulfur, obviously due to increase of their chemical stability and much lower sulfide toxicity at pH > 9 (**Figure 3a** and **Supplementary Figure S2**). Similar sulfidogenic activity was observed previously in cultures of natronophilic sulfur-reducing bacteria from soda lakes (Sorokin et al., 2010, 2011). The aerobic growth with acetate and SCFA was slow but resulted in a much higher biomass yield than during anaerobic growth. In the presence of yeast extract, aerobic growth resulted in the fastest growth rates and higher biomass yield (**Figure 3b**). In accordance with the isolation source, the soda lake isolates were obligate alkaliphiles with a much higher pH optimum (**Figure 3c**) compared to the moderate alkaliphilic strain AArc-Sl (**Supplementary Figure S3**). The salinity profiles for the growth of all strains were typical for extreme halophiles with a growth range from 2.5 to 5 M (optimum at 3.5–4 M), although different requirements of Cl^-^ were detected. The group of strains from soda lakes required only 0.5 M Cl^-^ for optimal growth, while AArc-Sl grew optimally at 3.5–3.8 M Cl^-^ (**Supplementary Figure S3a**). This indicated that the soda lake group behaves as typical natronophiles and AArc-Sl – as a haloalkaliphile. We also tested strain 93dlM4 for the ability to grow by sulfur reduction, since, despite being isolated as a heterotrophic aerobe, it was closely related to the group 1 AArc isolates. Indeed, after a period of 2–3 weeks lag phase, it grew anaerobically with elemental sulfur as acceptor using formate as *e*-donor and pyruvate or yeast extract as the C-source accumulating up to 30 mM sulfane at pH 9.8 and 4 M total Na^+^. On the other hand, neither *Nl. baerhaense*, nor *Nl. innermongolicus* showed no signs of anaerobic growth with sulfur with various electron donors positive for closely related AArc strains.

Washed cells of strain AArc1, grown with different *e*-donors and acceptors, were tested for the sulfur-reduction activity. We found that H_2_- and formate-dependent activities expressed differentially, while the cells grown with butyrate still expressed formate-dependent activity. Furthermore, the cells grown with DMSO were still active in sulfur reduction, but not vice-versa (i.e., cells grown with sulfur as acceptor were not active with DMSO). The aerobically grown cells did not express the sulfur-reducing activity (**Supplementary Figure S4**).

### Genomic Reconstruction of the Key Metabolic Features in Sulfur-Reducing Natronoarchaea

We determined complete genome sequences of the AArc1, AArc-Mg and AArc-Sl strains to gain a glimpse into metabolic versatility of this phenotypically novel group of sulfur-reducing natronoarchaea and to resolve their ecological role in anaerobic sulfur and carbon cycling in salt-saturated alkaline habitats. Most data are presented in **Supplementary Tables S2–S5** and **Supplementary Figures S5–S8**. As we discussed above, the first two strains are likely belonged to the new species within the genus *Natronolimnobius.* Both genomes comprised a circular chromosome of 3.5–3.6 Mb with a single 235 kbp plasmid pAArc-Mg-01 and two (101 and 166 kbp) plasmids pAArc1-01 and pAArc1-02, in strain AArc-Mg and strain AArc1, respectively (**Supplementary Table S2**). The genome of AArc1 contains 3470, 84 and 154 predicted protein-coding genes located in the chromosome and plasmids 1 and 2, respectively.

The AArc1 chromosome exhibited a high colinearity with the homologous replicon in AArc-Mg with few multiple inversions, genome rearrangements and the presence of CRISPR and CRISPR-associated elements in AArc1 genome are also found (**Supplementary Figures S6–S8**). The two complete CRISPR-Cas systems in AArc1^T^ compared to none in AArc-Mg emphasizes the eventual lifestyle differences of these isolates and past exposure to the lateral gene transfer, with AArc1 being a subject to a much stronger pressure by mobile genetic elements. A high degree of colinearity was also detected between the AArc1-2 plasmid and the AArc-Mg-1 plasmid (**Supplementary Figure S7**), while the pAArc1-1 plasmid had nothing in common with the entire AArc-Mg genome. The genome of AArc-Sl consists of a single 3,313,120 Mbp chromosome, with 3,232 annotated protein-coding genes, 2 rRNA operons, 47 tRNA genes, and two CRISPR regions. AArc1 and AArc-Sl showed no homology between the spacer sequences, likely implying a different history of phage interaction for the strains, which was anticipated based on their isolation from geographically distant locations (**Supplementary Figure S8**).

Besides the capacity to grow aerobically, the AArc-Sl strain represents a phenotypic counterpart to the HDA strains with restricted catabolic versatility, hence its genomic features were considered only for the phylogenetic and evolutionary comparisons of [Ni, Fe] uptake hydrogenase and molybdopterin oxidoreductases from the complex iron-sulfur molybdoenzyme (CISM) superfamily. Additionally, since genomes of AArc1 and AArc-Mg strains are very similar to each other, we further discuss only genomic features of the type strain AArc1.

### AArc1 Proteome Expression Profiling

The full consensus of AArc1 proteins expressed during aerobic growth on butyrate and anaerobic growth on formate (with two *e*-acceptors) was produced (**Supplementary Figure S9** and **Supplementary Data Set S1**). Aerobic growth on butyrate and anaerobic growth on formate and DMSO recruited the expression of more than 60% of chromosomal genes and one-third of all plasmid-located genes. Sulfur respiration with formate demanded much lower numbers of proteins: only 40% of chromosomal and 23% of plasmid-encoded proteins were expressed. As expected, highly abundant proteins were those involved in housekeeping functions, central metabolic pathways, electron transfer and energy production. A special study could be devoted to the detailed proteomic analysis of AArc1 grown under three different conditions; while here we used these data mainly for quantification of those proteins central for validation of our assumptions regarding the mechanisms underlying the physiology of this phenotypically novel archaea.

### Central Metabolism Reconstruction

As we demonstrated previously, the strictly anaerobic acetotrophic HAA can use acetate both as an electron donor and a carbon source but cannot respire with formate and hydrogen, as the lithoheterotrophic HDA does. In turn, the HDA strains lack the reaction sequences for the conversion of acetyl-CoA to cellular building blocks and are thus incapable of acetate assimilation (Sorokin et al., 2016a, 2017b). The facultatively anaerobic sulfur-reducing natronoarchaea, described in this study, represent a physiologically novel ecotype of sulfur-respiring haloarchaea, which is to some extent a mixture of those of previously characterized groups. Although acetate could not be used anaerobically as the electron donor for sulfur respiration, the AArc strains were capable of utilizing this substrate as a carbon source. Assimilation of acetate or other acetyl-CoA-generating substrates (peptone, yeast extract and amino acids) both aerobically and anaerobically requires the dedicated anaplerotic pathways for conversion of acetyl-CoA into cellular building blocks. Recently it was shown that in addition to the classical glyoxylate cycle, nearly 40% of all sequenced haloarchaea possess the novel methylaspartate cycle (MAC), where acetyl-CoA and oxaloacetate are transformed into the citric acid cycle (TCA) intermediates (Khomyakova et al., 2011; Borjian et al., 2016, 2017).

**FIGURE 4:**
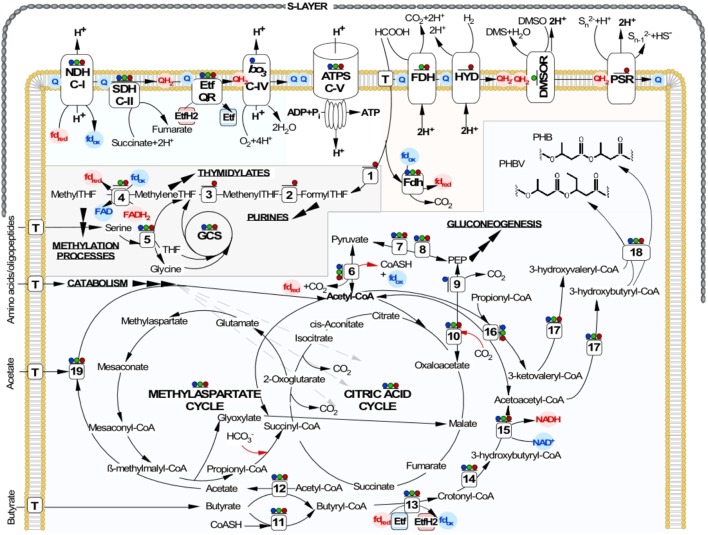
Reconstruction of central metabolic pathways, energy generation and proton-translocation machinery shared by NLS strains. ATP synthesis by chemiosmotic coupling of proton transport via aerobic and anaerobic respiratory chains is shown at the top. Below, the C_1_ metabolism, TCA and MAC cycles, as well as the SCFA oxidation and the PHA synthesis pathways are indicated. Inorganic carbon fixation is shown by red arrows. Presence of all enzymes in corresponding proteomes is shown as colored dots above: aerobic growth on butyrate – blue dots; anaerobic growth on formate (electron donor) and yeast extract (carbon source) with DMSO (electron acceptor) – green dots; with elemental sulfur (electron acceptor) – red dots. Enzymes involved: ATPS, archaeal ATP synthase; NDH C-I, Complex I NADH/ferredoxin dehydrogenase (ubiquinone); SDH C-II, Complex II succinate dehydrogenase; *bo_3_* C-IV, Complex IV cytochrome *bo_3_* ubiquinol oxidase, FDH, formate dehydrogenase; HYD, uptake hydrogenase; DMSOR, DMSO reductase; PSR, polysulfide reductase; Etf, electron transfer flavoprotein; Etf QR, electron transfer flavoprotein quinone reductase; GCS, glycine cleavage system; T, corresponding transporters; (1) formate—tetrahydrofolate ligase; (2) 5,10-methylenetetrahydrofolate reductase; (3) methylenetetrahydrofolate dehydrogenase; (4) methylenetetrahydrofolate reductase; (5) serine hydroxymethyltransferase; (6) pyruvate:ferredoxin oxidoreductase; (7) pyruvate kinase; (8) PEP synthase; (9) PEP carboxykinase; (10) PEP carboxylase; (11) acyl-CoA synthase; (12) acyl-CoA:acetate/3-ketoacid CoA transferase; (13) acyl-CoA dehydrogenase; (14) Enoyl-CoA hydratase; (15) 3-hydroxyacyl-CoA dehydrogenase; (16) acetyl-CoA acetyltransferase; (17) 3-ketoacyl-ACP reductase; (18) PHA synthase; (19) acetyl-CoA synthetase.

In agreement with cultivation results, genome analysis of the AArc strains identified the genes that are implicated in key reactions of biomass production from the acetyl-CoA donors. Unlike the acetoclastic HAA, which possesses the glyoxylate cycle (Sorokin et al., 2016a), the MAC is fully operational in the AArc strains (**Figure 4**) and all enzymes of this anaplerotic pathway together with those from the TCA are present in proteomes (**Supplementary Data Set S1** and **Supplementary Table S5**). In conformity with the capability of the AArc strains to grow on peptone and yeast extract as carbon and energy source, the genome analysis indicates that these organisms can efficiently uptake and consequentially convert proteins and peptides into amino acids. More than 30 cytoplasmic and membrane-associated proteases and aminopeptidases were found with similar numbers of ABC-type oligopeptide and amino acid transport systems. The well-developed capacity for amino acids degradation was also confirmed: alanine, glutamate and threonine dehydrogenases, anthranilate synthase, arginase, aspartate aminotransferase, histidinase and urocanase are just few examples of them.

Growth on amino acids typically requires the gluconeogenic pathway for carbohydrate synthesis (Danson et al., 2007) and in line with that all genes for a reverse glycolytic pathway, including pyruvate:ferredoxin oxidoreductase and archaeal fructose 1,6-bisphosphatase have been identified. Notably, the latter is a strictly anabolic enzyme, which is discussed as being an ancestral enzyme type (Say and Fuchs, 2010). Although AArc strains were unable to use any of the tested sugars as the sole carbon source, the genes coding for fructose 1,6-bisphosphate aldolase and some essentially irreversible reactions of glycolysis, e.g., archaeal ADP-dependent phosphofructokinase/glucokinase, are present in the genome. Together with fructose 1,6-bisphosphatase these genes are encoded by the gene cluster AArc1_2551-3. Thus, glucose can likely be converted into phosphosugars and pentoses if synthesized *de novo* by AArc cells. Consistently with findings in other halophilic archaea (Mwirichia et al., 2016), the oxidative pentose phosphate pathway is missing and pentoses are metabolized non-oxidatively by conversion of fructose 6-phosphate to ribulose 5-phosphate. Another source of the ribulose phosphates could be ribose sugars via the nucleotide salvage pathway (Sato et al., 2007). The three enzymes required in this archaeal pathway (AMP phosphorylase, ribulose-1,5-biphosphate synthetase and archaeal form III of RuBisCO) were identified in the AArc genomes.

Both AArc1 and AArc-Mg contained multiple genes encoding paralogs for the bacterial-like fatty acid β-oxidation pathway. Such abundance of beta-oxidation proteins reflects the metabolic capability of AArc strains from the group 1 to use C_4_-C_9_ SCFA as a single source of carbon and energy. Despite that paralogous genes are present in many other haloarchaea, reports on their ability to metabolize fatty acids are scarce and concern only a few examples of aerobic degradation of long chain fatty acids (Falb et al., 2008). On the other hand, the C_16_-fatty acid palmitate was shown to be crucial for the functional integrity of halorhodopsin. *Halobacterium salinarum* cells appear to be capable of synthesizing this long-chain fatty acid from C_2_-C_3_ precursors. Based on this observation, the β-oxidation pathway working in the reverse direction was proposed to catalyze the biosynthesis of fatty acids (Dibrova et al., 2014). Most of the enzymes required to convert butyrate into two molecules of acetyl-CoA, are scattered across the AArc1 genome, except those grouped in the chromosomal gene cluster AArc1_0659-0667 and in the pAArc1-02 plasmid cluster AArc1_5057-74. Gene organization in these clusters is similar to that found in many butyrate-degrading sulfate-reducing and syntrophic eubacteria. The butyrate oxidation in the AArc1 cells likely starts with the activation of substrate by either AMP-forming acyl-CoA synthase (6 paralogs) or acyl-CoA:acetate transferase (gene products of AArc1_0666 and AArc1_3042-3) (**Figure 4**). Subsequent transformation of butyryl-CoA proceeds via the classical pathway of fatty acids β-oxidation, terminating with the formation of two acetyl-CoA molecules. The β-oxidation of SCFA with odd carbon atoms requires a specialized pathway, which converts formed propionyl-CoA into methylmalonyl-CoA and then to succinyl-CoA. All these gene products (propionyl-CoA carboxylase and methylmalonyl-CoA mutase) are a part of the anaplerotic MAC pathway and are present in AArc genomes in multiple copies (**Supplementary Table S5**). Enzymes involved in MAC and SCFA metabolism were among the most abundant proteins detected under all conditions of AArc1 cultivation (**Supplementary Data Set S1**), indicating that these processes are the major catabolic systems and are substantially important for sustaining the life of this novel group of natronoarchaea.

Seasonal changes strongly affect microbial life in salt lakes. In particular, dramatic changes in salinity were observed in the soda lakes of the Kulunda Steppe and also seasonal temperature fluctuation. Such fluctuations might trigger both substantial change in dominating prokaryotic populations and the adaptive responses inside the same population, such as the switch from feast to famine. Haloarchaea may accumulate substantial amounts of polyhydroxyalkanoates (PHA) as energy and carbon storage polymers (Han et al., 2010). The AArc strains were not an exception, and the PHA synthesis has been detected under both aerobic and, to a lesser degree, anaerobic conditions of cultivation on butyrate (**Supplementary Figure S1**). The 3-ketoacyl-CoA reductase and PHA synthetase, which plays a key role by catalyzing the polymerization of (*R*)-3-hydroxyalkanoyl-CoA into PHAs, has been identified in one cluster (AArc1_1497-9) in the NLS genome. Similarly with known haloarchaeal counterparts, the AArc PHA synthetase comprises two subunits (PhaC and PhaE) and belongs to the archaeal subtype IIIA. The AArc1_1497-9 proteins were detected in all growth conditions indicating the importance of PHA synthesis during both aerobic and anaerobic cultivation of AArc cells. It is well documented that PHA accumulation could have a dual function in cell metabolism and be implicated as both an energy and carbon source and as an electron sink for the reducing power, which accumulates when electron flow through the electron transfer chain is compromised or arduous (Senior and Dawes, 1971, 1973). This second function of PHA synthesis seems essential for the anaerobic oxidation of butyrate. As it is discussed below, the butyryl-CoA/crotonyl-CoA redox couple is thermodynamically unfavorable; thence the reductive step in PHA synthesis could be advantageous while serving as an additive electron sink for the reducing power.

### Energy Metabolism Reconstruction

Genomic analyses of both AArc groups (**Supplementary Data Set S2**) indicated that their aerobic respiration likely proceeds via a membrane-bound electron transport chain (Complex I, ferredoxin: quinone oxidoreductase and Complex II, succinate: quinone oxidoreductase) that is terminated in the heme-copper containing terminal oxidases (Complex IV). All natronoarchaeal genomes lack genes encoding the Complex III (quinol: cytochrome *c* oxidoreductase), its alternative homologues (Refojo et al., 2010) and genes encoding for the *c*-type cytochromes. The lack of cytochrome *c* in cell-free extracts of strain AArc1 was confirmed by absorption spectra analysis (**Supplementary Figure S10**). All these findings indicated that AArc Complex IV should belong to the quinol-type of terminal oxidases, which are capable of using menaquinols as the electron donors. Confirming this, both *b*-type cytochrome and the genes encoding two terminal quinol oxidases, cytochrome *ba_3_* and cytochrome *bo_3_* were identified (**Supplementary Table S5** and **Supplementary Figure S10**). Both enzymes have four subunits and were encoded by *cba*DBAC (AArc1_3321-4) and *cyo*ABCD (AArc1_1310-3) operons, respectively. Direct oxidation of quinol conserves less energy than its oxidation by the *bc1* Complex III followed by cytochrome *c* oxidation by cytochrome *c* oxidases. Nevertheless, according to the Q-cycle mechanism, quinol oxidation by *ba_3_* and *bo_3_* terminal oxidases is of an advantage when quinone is completely reduced to quinol, because the cytochrome *bc*_1_ complex requires the presence of quinone for its function, (Gao et al., 2012). Such situation might be expected to dominate in sulfur-respiring haloarchaea, since reduction of the low potential and insoluble electron acceptor, such as sulfur, is most probably a limiting step in the respiratory chain operating at conditions of over-reduction of the menaquinone pool.

Proteomic analysis of AArc1 cells grown under three different conditions revealed that cytochrome *bo_3_*–quinol oxidase was exclusively expressed in aerobic cultures (**Figure 4** and **Supplementary Data Set S1**). Surprisingly, the catalytic CbaA subunit of cytochrome *ba_3_*–ubiquinol oxidase was apparently expressed in all three tested growth conditions. Considering that cytochrome *bd* quinol oxidase, encoded in all 3 analyzed AArc genomes was never expressed, it might be speculated that *ba_3_* enzyme takes over a oxygen detoxification function of cytochrome *bd* during anaerobic growth of these strains. Other candidate proteins involved in oxidative stress protection and expressed similarly to CbaA included a cytochrome *c* peroxidase (AArc1_1100), catalase/peroxidase (AArc1_2205) and superoxide dismutase (AArc1_1169).

**FIGURE 5:**
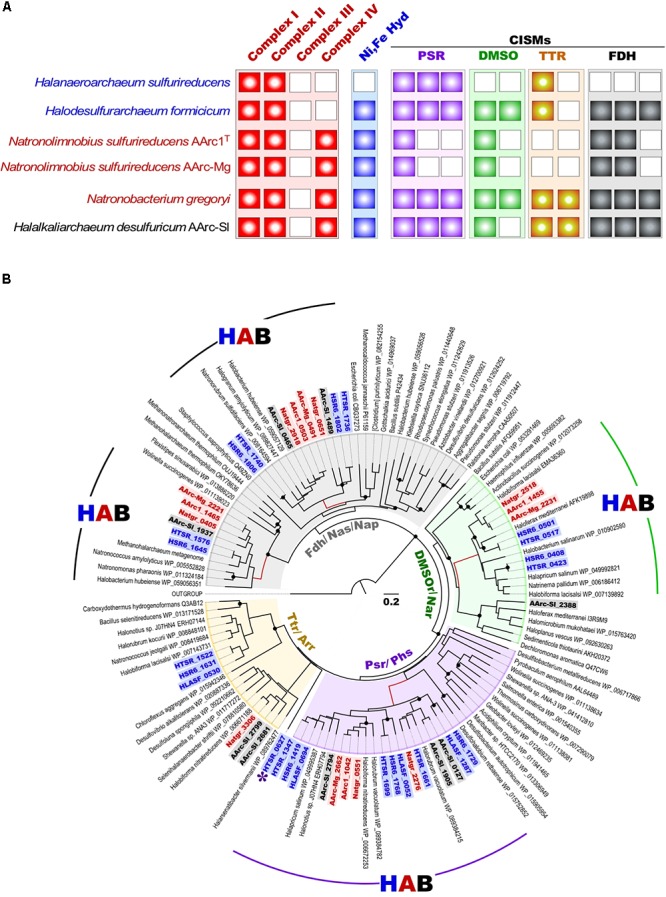
**(A)** Respiratory chain components in all experimentally proven sulfur-reducing haloarchaea; **(B)** Maximum Likelihood phylogenetic tree of CISM catalytic subunits A. Totally 118 sequences were taken for the CISM analysis. The tree with the highest log likelihood is shown. Locus tags of CISM proteins of sulfur-reducing members of the order *Halobacterales* (blue), *Haloferacales* (black) and *Natrialbales* (red) are highlighted in bold. Unknown CISM family is shown by asterisk. Abbreviations used: DMSO/Nar, DMSO/nitrate reductase family; Fdh/Nas/Nap/, assimilatory nitrate reductase/formate dehydrogenase family; HAB, Haloarchaeal Branch; HLASF, *Halanaeroarchaeum sulfurireducens* HSR2^T^; HSR6, *Halodesulfuriarchaeum formicicum* HSR6^T^; HTSR, *Halodesulfuriarchaeum formicicum* HTSR1; Natgr, *Natronobacterium gregoryi*; Ni, Fe Hyd, [NiFe] uptake hydrogenase; Psr/Phs, polysulfide/thiosulfate reductase family; Ttr/Arr, tetrathionate/arsenate reductase family. Scale bar is 0.2 amino acid substitutions per site. White boxes indicate that no homolog was detected **(A)**. Filled circles at the nodes indicate the bootstrap values > 70% (1000 bootstrap iterations).

As mentioned above, besides the capability for aerobic respiration, strain AArc-Sl exhibits the type of metabolism very similar to that of HDA (Sorokin et al., 2017b). In confirming this, its genome encodes an analogous set of nine molybdopterin oxidoreductases from the CISM superfamily (**Figure 5**). In contrast, the AArc1 and AArc-Mg set of CISM enzymes consisted of only two (membrane-bound and cytoplasmic) Fdh formate dehydrogenases and the solitary DMSO and polysulfide reductases (DMSOR and PSR), thus representing a “minimal” respiratory outfit, needed for formate-dependent respiration with DMSO and elemental sulfur. Such economic genetic potential makes the AArc group 1 strains the ideal model organisms for unambiguous identifying the essential machinery of observed types of respiration. Phylogenetic analysis of the CISMs, detected in the AArc group 1, revealed that together with other haloarchaeal counterparts, Fdh, DMSOR and PSR enzymes form deep branches within the corresponding CISM families and likely represent ancient forms of molybdopterin oxidoreductases (**Figure 5**).

In a like manner with formate-oxidizing HDA strains, to exploit the low reduction potential of this electron donor (E_0_‘[CO_2_/HCOO^-^] = -430 mV) two types of formate dehydrogenase (membrane-anchored and cytoplasmic) are engaged by sulfur-respiring natronoarchaea into coupling its oxidation to reduction of various electron acceptors. The first type of Fdh coded by an operon of three-subunits membrane-bound peripherally oriented formate dehydrogenase (AArc1_1462-5), which is expressed only anaerobically. Thus, this enzyme likely represents a part of anaerobic respiration chain and mediates transfer of electrons via a menaquinone pool to terminal reductases DMSOR or Psr. Cytoplasmic type of monosubunit Fdh (AArc1_0503) seems interact with the electron transfer flavoproteins EtfAB (AArc1_0509-10) to perform energy conservation by coupled reduction of ferredoxin and NAD^+^, as we recently proposed for HDA (Sorokin et al., 2017b).

**FIGURE 6:**
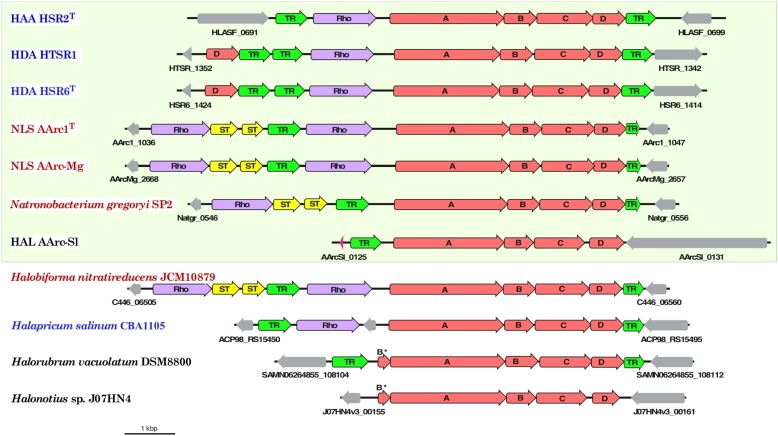
Organization of gene clusters encoding polysulfide reductase (Psr) in haloarchaeal genomes. Arrows show the direction of transcription. Bar represents scale of 1000 bp and the genes are drawn to scale. Organisms with proven capability of lithotrophic sulfur-reducing growth using hydrogen as the electron donor are boxed. The members of orders *Halobacteriales*, *Haloferacales* and *Natrialbales* are highlighted in blue, black and red, respectively. The genes associated with neither Psr reductase subunits (A-D, red) nor sulfur-transferases (ST, yellow), rhodanese-sulfurtransferase (Rho, violet) and transcriptional regulators (TR, green) are shown in gray colors.

Aforementioned presence of a single set of PSR polysulfide reductase genes in the group 1 AArc genomes unequivocally indicates that their products are responsible for sulfur utilization as terminal electron acceptor. This polycistronic operon comprises genes encoding MoPterin (*psr*A), FeS subunit (*psr*B), membrane quinol-interacting anchor protein (*psr*C) and system-specific chaperone (*psr*D). The presence of a TAT motif in the large MoPterin subunit indicates its export across membrane. In addition to the *psr*A-D genes, the AArc1 and AArc-Mg Psr operon includes genes encoding various types of sulfurtransferases and proteins of the bacterioopsin transcriptional activator family (**Figure 5**). Phylogenetic analysis of CISM catalytic subunits, encoded in the genome of AArc-Sl, revealed that the strain possesses three putative Psr operons, among which one does not have *psr*C (AArc-Sl_2794-6), while second operon was flanked by genes encoding proteins conferring resistance to arsenite (AArc-Sl_1902-5). Third Psr operon (AArc-Sl_0126-30) does not have adjacent sulfurtransferases genes, which are scattered across the genome (AArc-Sl_1380 and AArc-Sl_3093). Recently, we proposed that the rhodanese-like sulfurtransferase, which acts as a polysulfide-binding carrier, is likely responsible for sulfur mobilization during sulfur respiration in HAA and HDA strains (Sorokin et al., 2016a, 2017b). Their rhodanese-containing Psr operons show very similar gene composition to the AArc group 1 operons with a 35-68% amino acid identity of the reading frames (**Figure 6**). Proteomic analysis of AArc1 cells grown under three different conditions revealed that PSR and DMSOR reductases were exclusively expressed in anaerobic cultures with elemental sulfur or DMSO as acceptors, respectively (**Figure 4** and **Supplementary Data Set S1**). The fact, that the cells grown with DMSO were still capable of sulfur reduction, albeit with lower activity than those grown with sulfur (**Supplementary Figure S4**), might indicate that the DMSOR of AArc1 also has some Psr activity.

The results of bioinformatic inspection of currently available haloarchaeal genomes demonstrated that at least three haloarchaeal species, *Halapricum salinum*, *Halobiforma nitratireducens* (former *Natronobacterium nitratireducens*) and *Natronobacterium gregoryi*, most probably encode the genuine rhodanese-containing Psr operons. In addition, similarly to AArc-Sl, the genomes of *Halorubrum vacuolatum* and *Halonotius* sp. J07HN4 (all members of the order *Haloferacales*) have the Psr operon, lacking rhodanese-like sulfurtransferase genes, which are presumably spread across their yet unassembled genomes (**Figure 6**). Detailed analysis of the genomes of these five haloarchaea, the putative sulfur reducers, revealed that only *Natronobacterium gregoryi*, originally described as an obligate aerobic heterotroph (Tindall et al., 1984), contains all genomic determinants required for sulfur-reducing lithotrophic growth. Indeed, after two month-long period of adaptation to anaerobic conditions of the aerobically grown culture of *N. gregoryi* DSMZ 3393. Aerobically grown cells of *N. gregoryi* DSMZ 3393 after 2 months-long period of adaptation to anaerobic conditions, the genomic inference of this metabolic potential was, indeed, confirmed. Using the elemental sulfur as terminal electron acceptor, a strong sulfidogenic activity was detected during the anaerobic lithoheterotrophic growth of *N. gregoryi* with hydrogen (but not with formate) as an electron donor.

**FIGURE 7:**
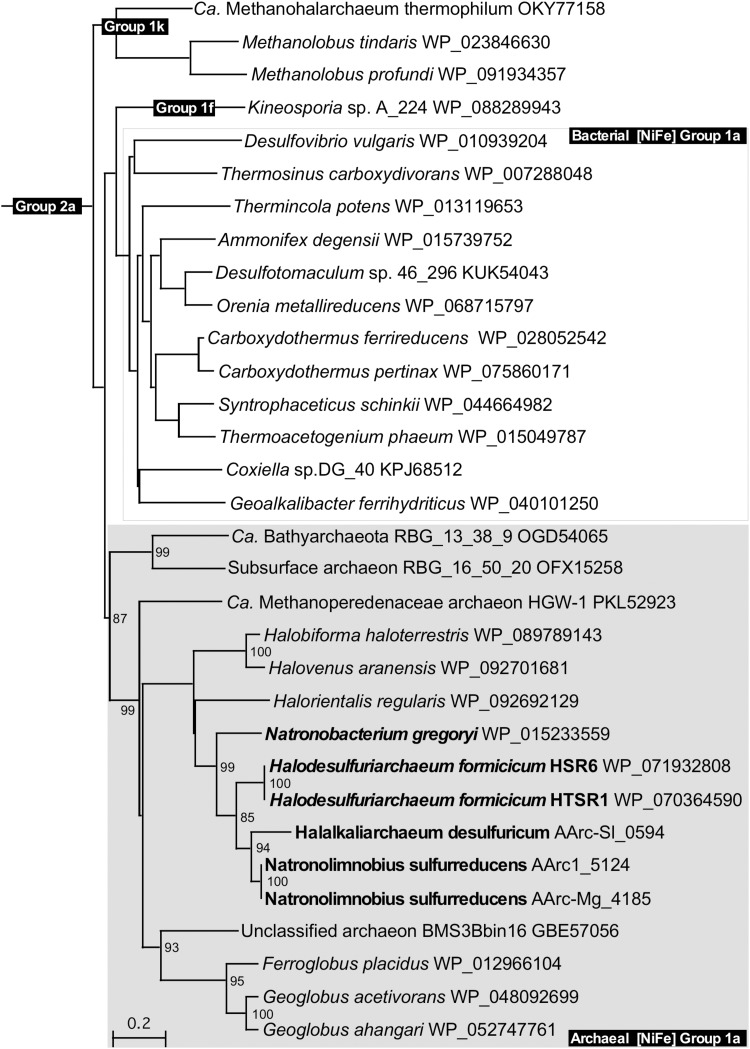
Phylogenetic tree of all haloarchaeal respiratory H_2_-uptake [NiFe]-hydrogenases constructed with full-length HydB subunits. The bootstrap values of more than 75%, supporting topological placement of haloarchaeal HydB subunits, are displayed as percentages close to the corresponding nodes. Members of the class *Halobacteria* with experimentally confirmed ability for lithotrophic growth with hydrogen are highlighted in bold. [NiFe] Group 2a-hydrogenases of *Ca. Syntrophoarchaeum caldarius* (GenBank accession no. OFV67551) was used as the out-group. Branch lengths along the horizontal axis reflect the degree of relatedness of the sequences (20%).

Physiological studies showed that both AArc groups gained energy from anaerobic hydrogen oxidation with elemental sulfur or DMSO (group 1) as terminal electron acceptors, pointing at their hydrogen-dependent lithotrophic lifestyle. Inspection of annotated genomes revealed in these natronoarchaea a solitary set of genes encoding the [NiFe] hydrogenase subunits including the small FeS subunit HydA (42.6 KDa), the large NiFe subunit HydB (56.3 KDa) and membrane-anchored cytochrome *b* subunit HydC (35.5 KDa). Of note, in both AArc1 and AArc-Mg strains, the [NiFe] hydrogenases genes are plasmid-encoded and located on pAArc1-02 and pAArc-Mg-01 plasmid, respectively. Analysis of the available haloarchaeal genomes showed that in addition to *N. gregoryi*, at least three other members of *Halobacteria* possess strikingly similar [NiFe] hydrogenases. Using the recently created web tool for hydrogenase classification (Søndergaard and Pedersen, 2016), all these enzymes can be classified as members of the respiratory H_2_-uptake and O_2_-sensitive Group 1a [NiFe] hydrogenases. It was unexpected, since this group of hydrogenases, widespread in sulfate-reducing *Deltaproteobacteria* and *Firmicutes*, currently includes only bacteria. Phylogenetic analysis of the full-length HydA subunit revealed that together with hydrogen-oxidizing Fe^3+^-reducing *Archaeoglobi* and some unclassified archaeons, the haloarchaeal [NiFe] hydrogenases are organized in a deeply branched and topologically robust cluster (**Figure 7**). The formation of an active hydrogenase requires a complex maturation process, including the incorporation of metal ions (Fe, Ni) and CO and CN ligands in the active center, the orientation of the Fe–S clusters within the small subunit HydA, and the proteolytic cleavage of the C-terminal end of the large subunit HydB by an endoprotease (Casalot and Rousset, 2001). In most H_2_-oxidizing bacteria and archaea, the maturation protease gene *hyd*D and the hydrogenase pleiotropic genes *hypA–hypF* coding for accessory proteins appear to be scattered along the chromosome (Maróti et al., 2003; Siebers et al., 2011). However, in the analyzed haloarchaeal genomes, both *hyd*D and *hyp*A–*hyp*F genes are adjacent to the structural [NiFe] hydrogenase *hyd*ABC operon (**Supplementary Figure S11**). Remarkably, some of these modules are additionally flanked by various Na-dependent and ABC-type uptake systems, including ATP-binding transport cassettes required for nickel/cobalt and iron acquisition. Such operons apparently represent a compact complete suite of all genetic determinants of hydrogenase maturation and hydrogen oxidation, suitable for lateral gene transfer.

Although the evolutionary issues are out of the scope of the present study, the unprecedented uniformity of gene arrangements in haloarchaeal Psr and Hyd genomic suites/cassettes, highly conserved in members of all three orders of class *Halobacteria*, raises the question whether those were inherited from the Haloarchaeal Common Ancestor (HACA) and if so, whether HACA was capable of lithotrophic growth on hydrogen and sulfur before the first build-up of free oxygen in the atmosphere and before the mass acquisition of eubacterial genes, which hypothesized as to cause the radical physiological transformation of majority of haloarchaea to obligate aerobic heterotrophs (Nelson-Sathi et al., 2012).

Of a special interest is the anaerobic growth of the group 1 AArc strains on the non-fermentable organic compounds including C_4_–C_9_ SCFA, as the electron donor and carbon source during elemental sulfur reduction. This type of respiration is well studied in dissimilatory sulfidogens and syntrophic bacteria (McInerney et al., 2007; Stams and Plugge, 2009; Kuever et al., 2014; Worm et al., 2014). Energy conservation via this pathway includes two oxidative reactions that generate electron pairs and one substrate-level phosphorylation via phosphate acetyltransferase and acetate kinase. Generated ATP is partially invested in the endergonic conversion of butyryl-CoA to crotonyl-CoA (Schmidt et al., 2013). The studied natronoarchaeal sulfidogens lack this type of energy conservation, because neither phosphate acetyltransferase nor acetate kinase is present in their genomes. Apparently, formation of ATP from conversion of acetyl-CoA into acetate could be performed by acetyl-CoA synthetase (AArc1_1873), as it originally described in cellular extracts of acetate-forming Halobacterium saccharovorum (Schäfer et al., 1993). Substrate-level phosphorylation can be also expected via ATP-citrate lyase (AArc1_1994 and AArc1_2540 gene products), as described for *Desulfobacter* species (Rabus et al., 2006). The biochemical mechanism that enables the investment of a fraction of ATP for the endergonic conversion of butyryl-CoA to crotonyl-CoA has recently been revealed in butyrate-oxidizing *Syntrophomonas wolfei* (Müller et al., 2009; Schmidt et al., 2013; Worm et al., 2014; Sieber et al., 2015). Electrons that are generated by the conversion of butyryl-CoA to crotonyl-CoA travel via butyryl-CoA dehydrogenase, an electron transfer flavoprotein (EtfAB) and a membrane-anchored EtfAB:menaquinone oxidoreductase to the menaquinone pool in the membrane. Inspection of the AArc group 1 genomes delineated a similar conduit for electron transfer between acyl-CoA dehydrogenases and membrane redox carriers. This system likely includes the EtfAB complex (AArc1_1644-5 and AArc1_5075-6 gene products) and membrane-bound, electron transfer flavoprotein (EtfAB):menaquinone oxidoreductase (AArc1_1643 and AArc1_3420). Adjacent to the AArc1_1643-5 EtfAB complex, the AArc1_1630-3 gene products are organized in a system, structurally similar to the electron-bifurcating ubiquinol reductase FixABCX complex of *S. wolfei*. The putative Fix complex consists of FixAB, which seems to be an analog of the EtfAB3 complex (AArc1_1633 and AArc1_1632 gene products), FixC, an Etf:quinone oxidoreductase (AArc1_1631 gene product) and FixX, a ferredoxin (AArc1_1630 gene product). Recently it was proposed, that in association with the Bcd-EtfAB complex, the FixABCX system could participate in electron transfer and bifurcate two electrons to reduce both menaquinone and ferredoxin (Herrmann et al., 2008; Sieber et al., 2015; Ledbetter et al., 2017; Buckel and Thauer, 2018). Thus, in addition to functioning as a catabolic system during anaerobic growth on butyrate, the FixABCX complex (AArc1_1630-3) could supply reduced ferredoxin for biosynthetic purposes, e.g., pyruvate synthesis from acetyl-CoA and CO_2_ (Sieber et al., 2015). This assumption seems very plausible, since the genes encoding for pyruvate:ferredoxin oxidoreductase (AArc1_1638-40) are adjacent to identified EtfAB and FixABCX complexes.

## Conclusion

Extremely halophilic Euryarchaeota are the predominant group of microorganisms thriving in large areas across the planet where salt concentrations approach the saturation level and have long been considered important for understanding early microbial evolution (Nelson-Sathi et al., 2012; Groussin et al., 2015; Spang et al., 2017). Despite their phylogenomic proximity to strict anaerobic methanogens, until recently, the majority of haloarchaea were considered as obligate heterotrophs that use O_2_ as the terminal acceptor of their electron transport chain. The ability of alkaliphilic natronoarchaea to respire elemental sulfur and DMSO during growth on hydrogen, formate and with C_4_–C_9_ SCFA indicates that this type of respiration is distributed not only among neutrophilic haloarchaea. This suggests that the metabolic versatility and the contribution of halophilic Euryarchaeota to global carbon and sulfur cycling may be much greater than previously envisaged.

## Author Contributions

DS performed sampling and filed measurements, sediment incubations and isolation, and physiological test of pure cultures. EM, VLC, and MY performed phylogenetic and genomic analyses. MF, SC, and MM performed proteomic analysis. EM and ST sequenced and annotated the genomes. DS, MY, and PG wrote the manuscript.

## Conflict of Interest Statement

The authors declare that the research was conducted in the absence of any commercial or financial relationships that could be construed as a potential conflict of interest.

## Footnotes

1 http://www.genome.jp/tools/blast/
