# Supplementary material for: Sulfur Respiration in a Group of Facultatively Anaerobic Natronoarchaea Ubiquitous in Hypersaline Soda Lakes
Source: Front Microbiol. 2018 Oct 2;9:2359. doi: 10.3389/fmicb.2018.02359 (PMC6176080; doi:10.3389/fmicb.2018.02359)
Supplement: Supplementary file 1 [file Data_Sheet_1.doc]

**SUPPLEMENTARY INFORMATION**

**Sorokin et al.**

**Sulfur respiration in facultatively anaerobic natronoarchaea ubiquitous in hypersaline soda lakes.**

**Contents Page**

**Supplementary Figures S1 to S11 2-11**

**Supplementary Tables S1 to S5 12-14**

**Supplementary Data and associated references 15-16**

**SUPPLEMENTARY FIGURES**


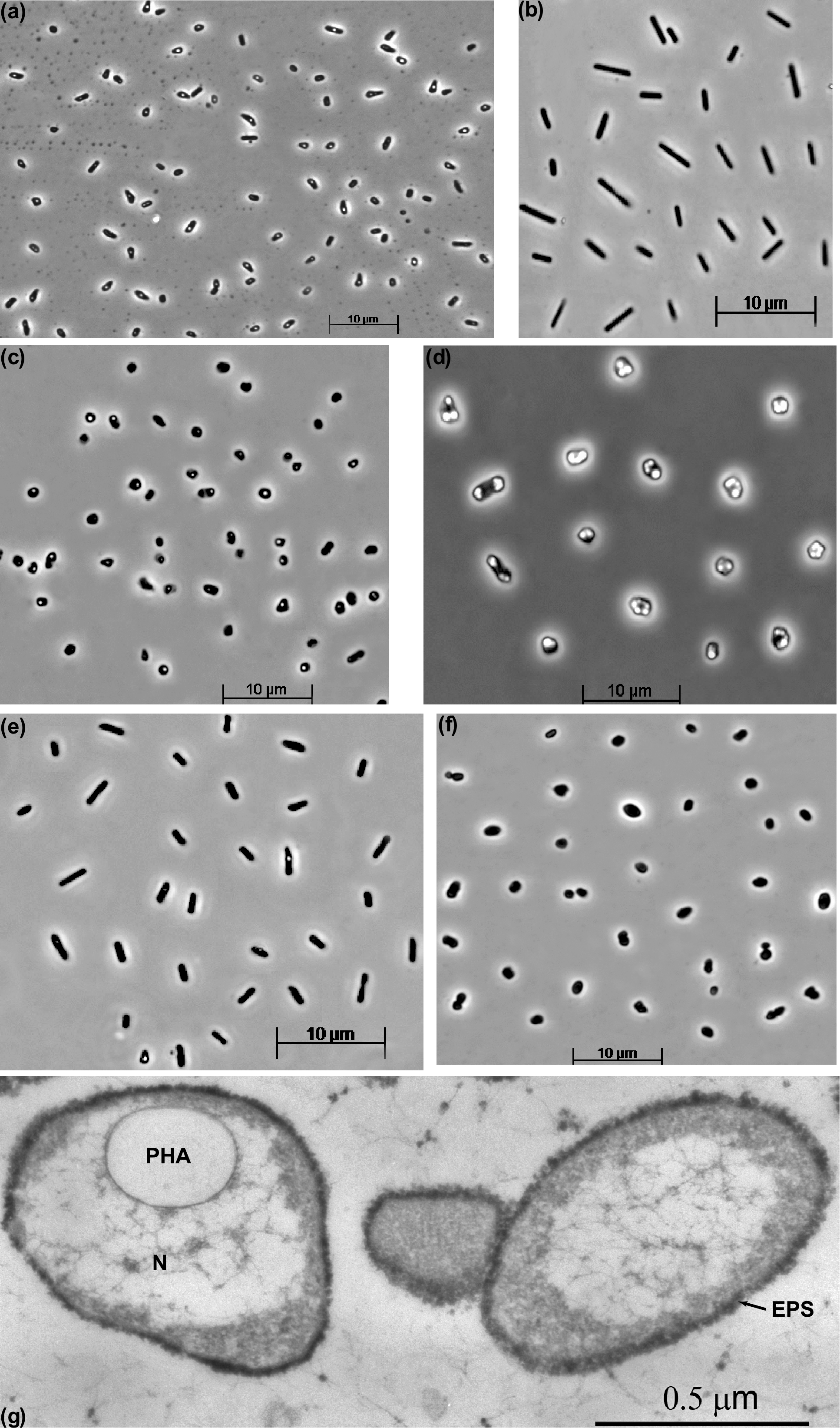


**Figure 1.** Cell morphology and ultrastructure of sulfur-reducing natronoarchaea. Phase contrast microphotographs: (**a, b**) strain AArc1 grown anaerobically with formate+S_8_ and formate+DMSO, respectively; (**c, d**) strain AHT32 grown anaerobically with butyrate+S_8_ and micro-aerobically with butyrate/yeast extract, respectively; (**e, f**) strain AArc-Sl grown anaerobically with formate+S_8_ and aerobically with pyruvate/yeast extract; (**g**) thin section electron microphotograph of cells of strain AArc1 grown anaerobically with butyrate+S_8_ shows PHA granule, extended nucleoid (N) and the EPS layer.


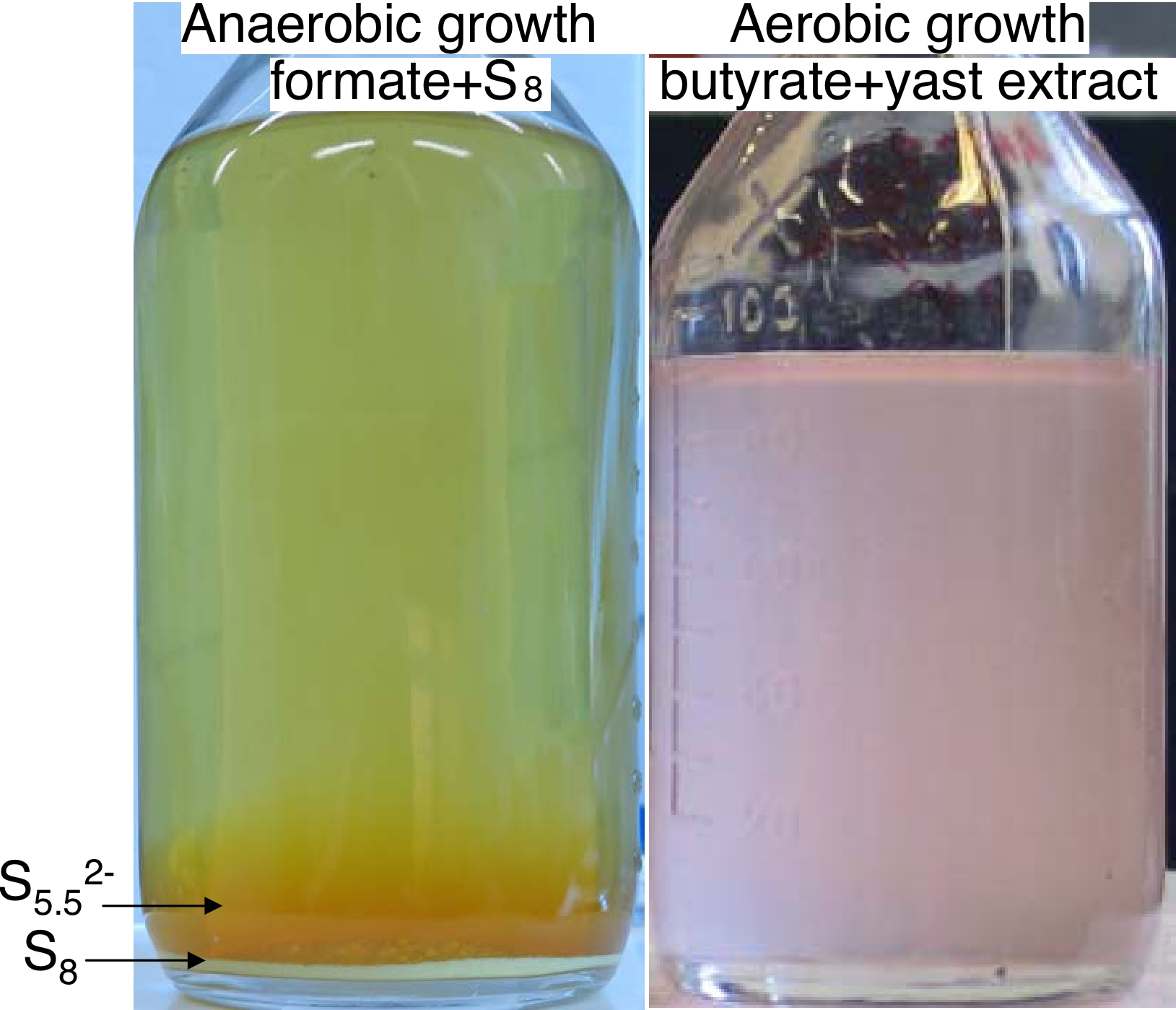


**Figure S2** Sulfur-reducing and aerobic cultures of strain AArc1^T^ grown at 4.0 M total Na+, pH 9.8 and 37°C. In anaerobic culture the crystalline sulfur is lying on the bottom of the flask. The initial microbial reduction of sulfur results in formation of HS^-^, which, in turn, is chemically reacting with the remaining sulfur and forms polysulfides as a stable intermediate. The reaction is taking place near the sulfur-containing layer at the bottom of cultivation flask. Then polysulfide is likely start to act as the actual electron acceptor for anaerobic growth.

**Figure S3.** Cultivation of sulfur-reducing natronoarchaeon AArc-Sl^T^. (**a**) Influence of pH at 4.0 M total Na^+^ on anaerobic growth with formate and sulfur and aerobic growth with peptone / yeast extract; numbers in parentheses indicate the total Cl^-^ concentrations. (**b**) Influence of salinity at pH 9.1 on aerobic growth with pyruvate and yeast extract. The data are mean values from 2 replicate cultures.


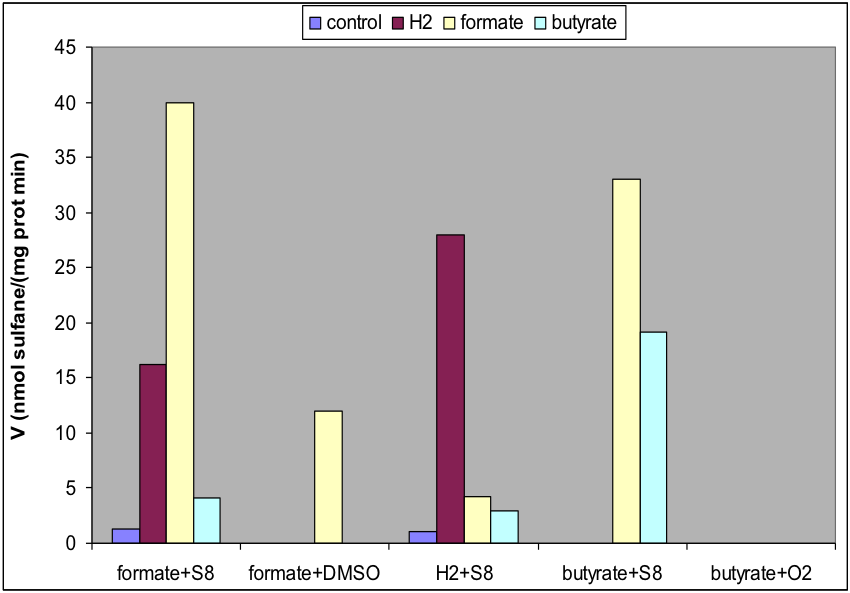


**Figure S4**. Sulfur-reducing activity of washed cells of AArc1 (at 4 M total Na+, pH 9.5, 37^o^C) with different electron donors depending on the growth conditions indicated on the X axis. The cells grown with formate + sulfur did not show any activity of DMSO reduction. "control" means no electron donor addition. The data are mean values from 2 replicates. Incubation time was 48 h.

**Figure S5.** Complete genomic maps of AArc1^T^, AArc-Mg and AArc-Sl^T^.


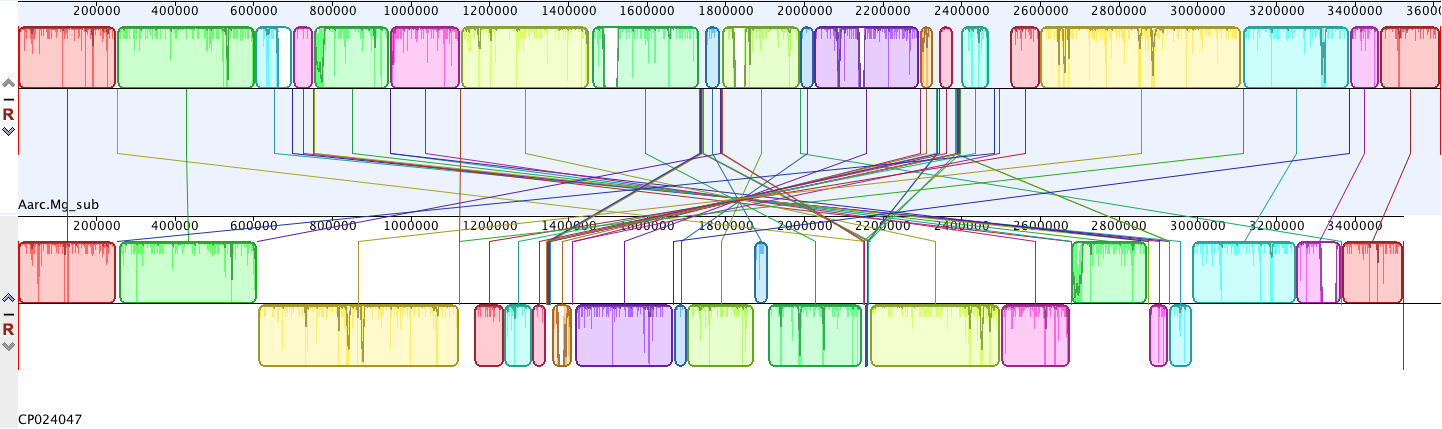


**Figure S6** Mauve genome alignment between AArc1^T^ and AArc-Mg chromosomes.


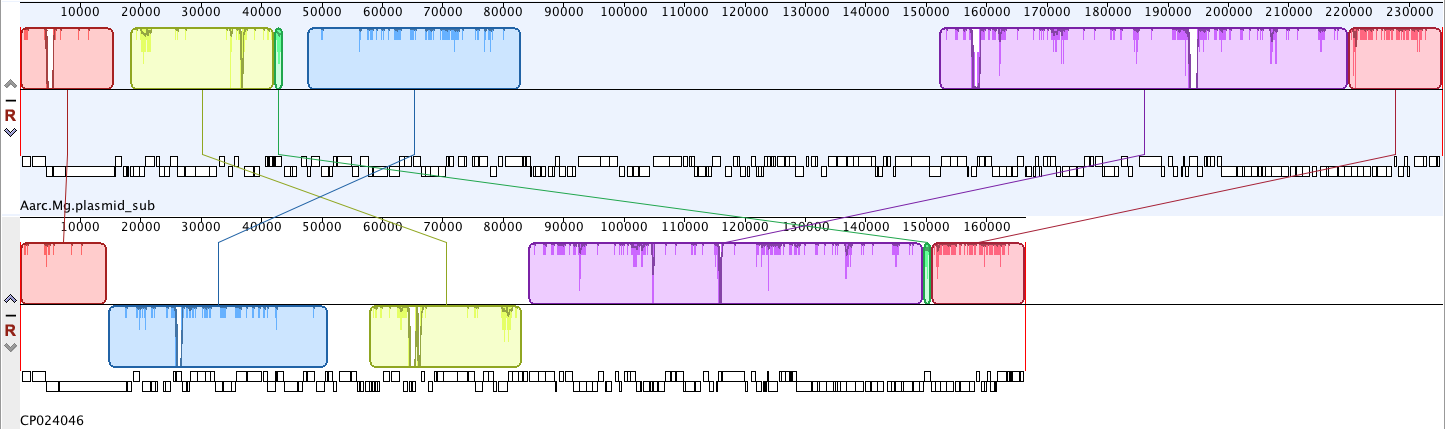


**Figure S7** Mauve genome alignment between pAArc1-02 and pAArc-Mg-01 plasmids.

**Figure S8** Structure of CRISPR-Cas systems identified in AArc1^T^ and AArc-Sl^T^ genomes.

**
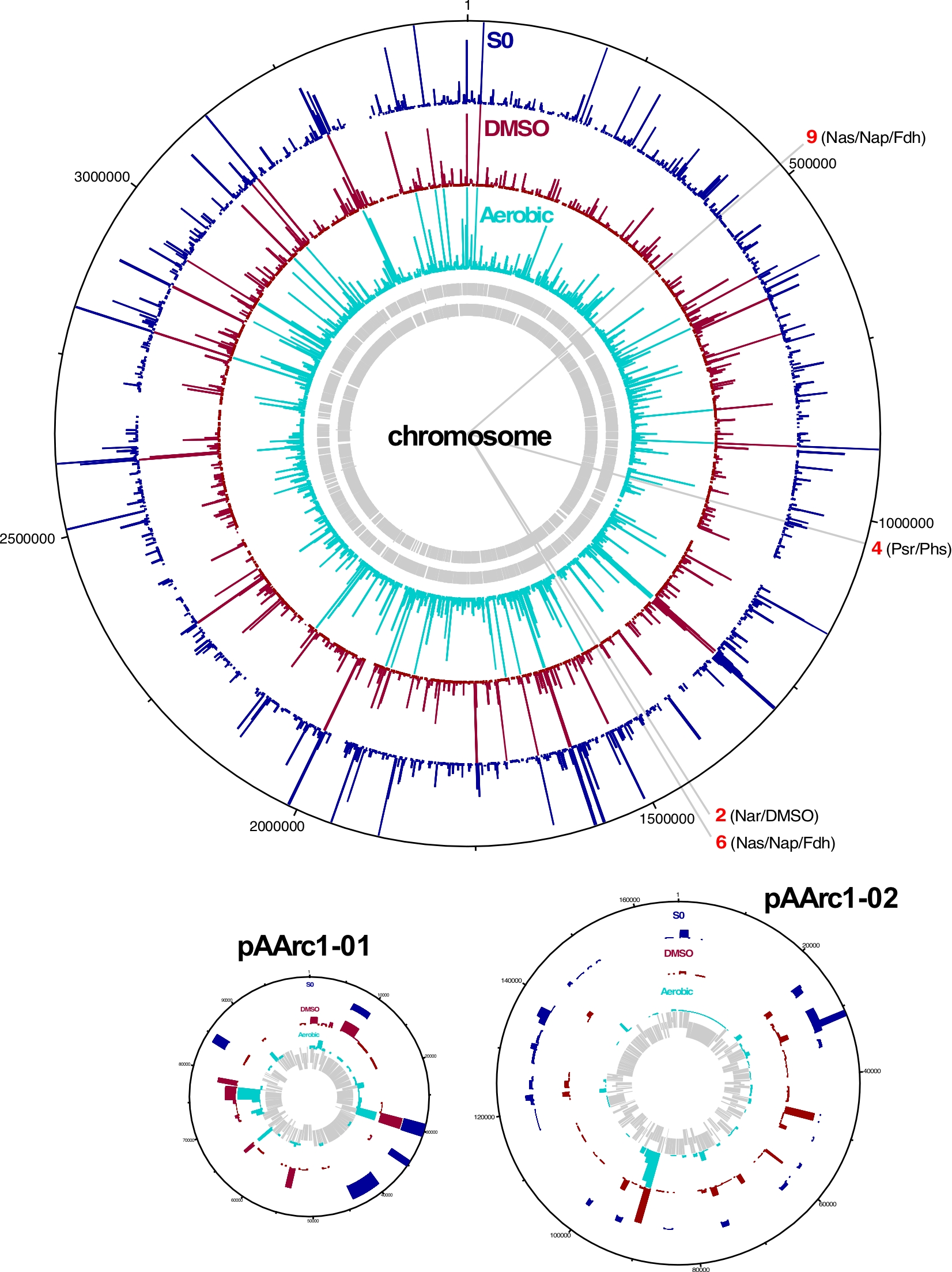
**

**Figure S9** Schematic map of differential protein expression under three cultivation conditions along the AArc1^T^ genomic contigs. The outermost ring indicates the position on the genome map of the CISM enzymatic complexes numbered following the HDA CISM complexes: DMSO reductase (2), polysulfide reductase PSR (4), and two formate dehydrogenases FDH (6, 9). The second, third and fourth rings (histograms) are normalized (nemPAI) values, which were obtained from the emPAI values by dividing each individual value by the sum of all emPAI values in a given experiment. Aerobic cultivation with butyrate, anaerobic cultivation with formate + DMSO and anaerobic cultivation with formate + elemental sulfur are colored in cyan, magenta and blue, respectively. Two innermost grey rings indicate predicted ORFs on the plus and minus strands, respectively.


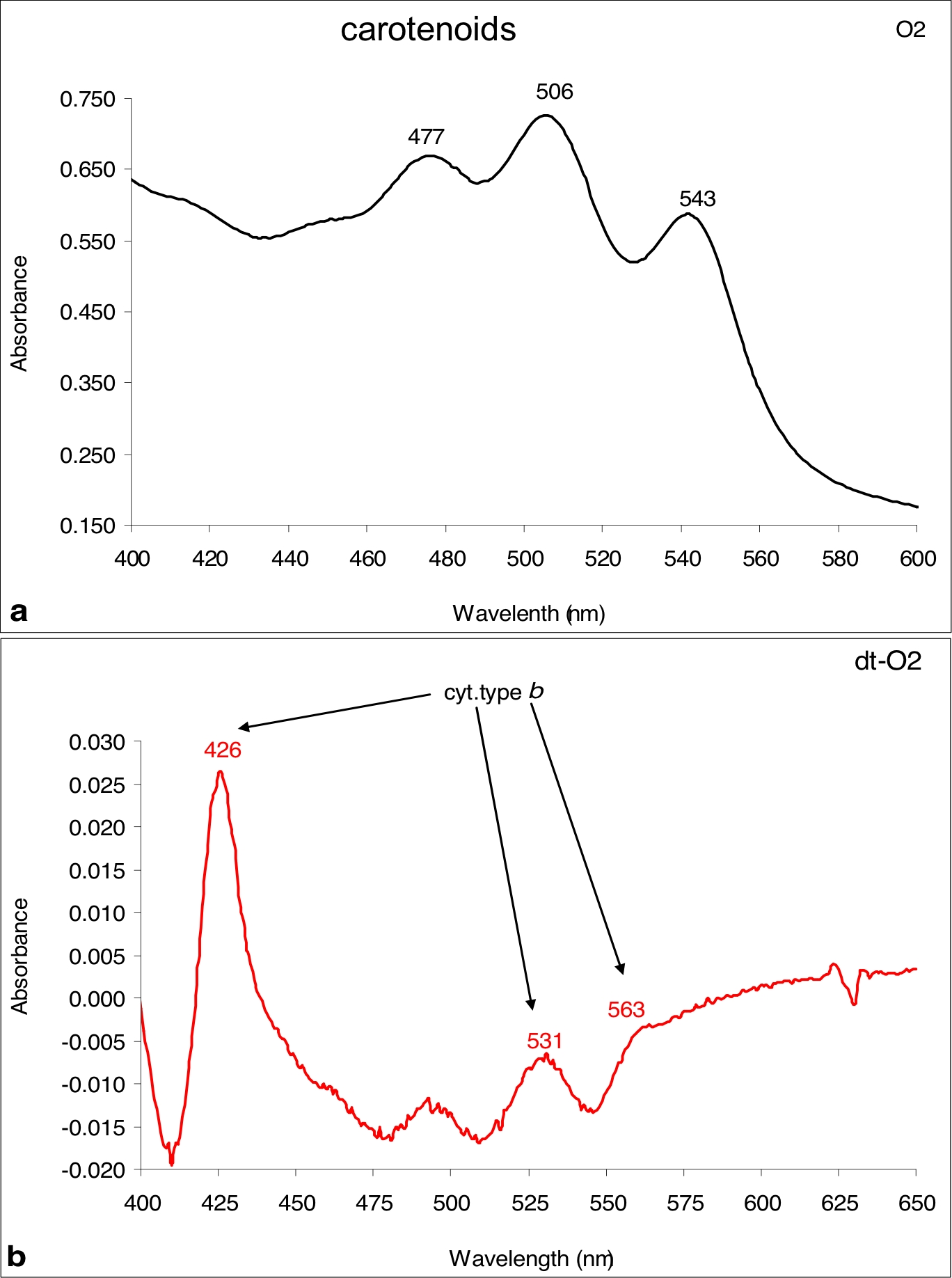


**Figure S10** Absorption spectra in cell-free extracts of strain AArc1T grown aerobically with butyrate. **(a)** Oxidized spectrum shows three picks typical for bacterioruberin; *(b)* dithionite-reduced spectrum demonstrates a presence of *b*-type cytochrome.


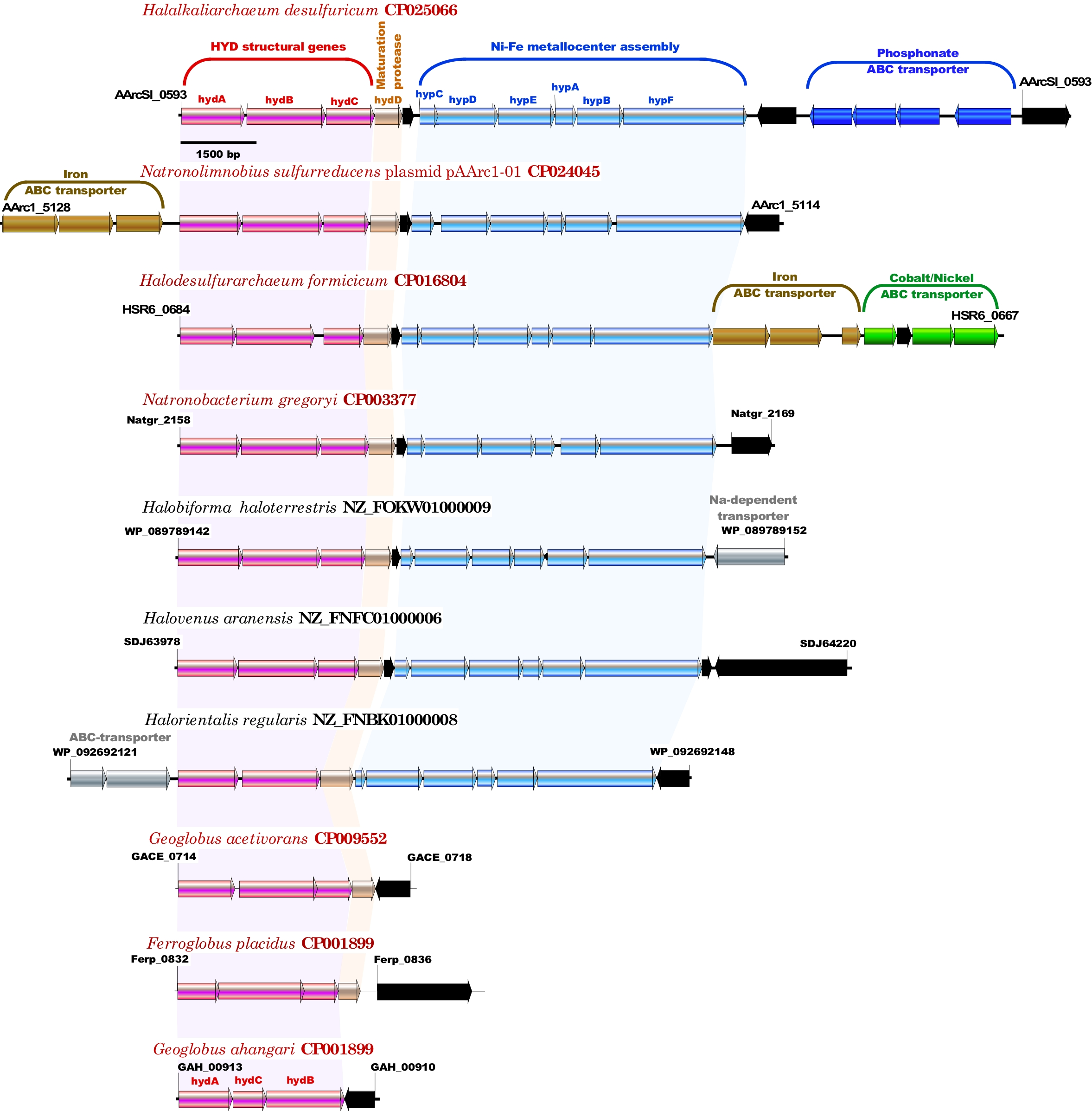


**Figure S11** Organization of the archaeal genomes around the genes encoding [NiFe] hydrogenase (including accessory genes). Arrows show the direction of transcription. Bar represents scale of 1500 bp and the genes drawn to scale. The organisms with proven capability of lithotrophic anaerobic growth using hydrogen as the electron donor are highlighted in red. The genes, neither associated with hydrogenase nor uptake systems, are shown in black.

**SUPPLEMENTARY TABLES**

**Table S1** Key chemical characteristics of hypersaline soda and alkaline lakes, brine and sediments from which were used as inoculum for isolation of sulfur-reducing natronoarchaea.

| Lake | Location | | Total salts  (g/l) | pH | Soluble carbonate  alkalinity (M) |
| --- | --- | --- | --- | --- | --- |
|  | Area | Coordinates |  |  |  |
| Cock Soda Lake | Kulunda Steppe  (Altai, Russia),  2010-2012 | N52^o^16'/E79^o^52' | 100 | 10.1 | 0.7 |
| Tanatar-1 |  | N51^o^39'/E79^o^48' | 400 | 11.0 | 5.0 |
| Bitter-1 |  | N51^o^40'/E79^o^54' | 330 | 10.3 | 4.0 |
| Trona crystallizer |  | N51^o^39'/E79^o^46' | 380 | 9.6 | 3.1 |
| Picturesque Lake |  | N51^o^43'/E79^o^52' | 250 | 9.5 | 2.8 |
| Stamp Lake |  | N51^o^41'/E79^o^46' | 240 | 9.1 | 0.15 |
| Shar-Burdiin, Hotontyn | north-eastern Mongolia, 1999 | N48^o^50'/E113^o^51' | 220-360 | 9.6-9.9 | 0.9-1.2 |
| Lake Badain | Badain-Jaran desert^a^  (Inner Mongolia), 2013 | N39º33'/E102º21' | 495 | 9.7 | 1.4 |
| Hamra, Fazda, Beidah, Ruzita,  Zugm, Khadra, Umm-Risha, Gaar | Wadi al Natrun ( Egypt) 2000 | N30º24'/E30º18' | 200-360 | 9.1-9.9 | 0.1-0.9 |
| Searles Lake | California, 2005^b^ | N35º44'/W117º20' | 350 | 9.8 | 0.2 |
| Owens Lake | California, 2008 | N36º26'/W117º57' | 180 | 9.7 | 1.0 |

^a^ provided by Dr. Zorigto Namsaraev

^b^ provided by Prof. Ronald Oremland

**Table S2** General characteristics of the AArc genomes.

| Attribute | *N. sulfurireducens* AArc1^T^ | *N. sulfurireducens* AArc-Mg | *H. desulfuricum* AArc-Sl ^T^ |
| --- | --- | --- | --- |
| Genome composition | 1 chromosome  2 plasmids | 1 chromosome  1 plasmid | 1 chromosome  - |
| **Chromosome** size (G+C) | 3,521,804 bp (62.9%) | 3,617,932 bp (62.8%) | 3,313,120 bp (63.1%) |
| DNA coding region (%) | 3,001,016 bp (85.2%) | 3,072,329 bp (84.9%) | 2,927,283 bp (88.4%) |
| Total genes | 3,530 | 3,588 | 3,285 |
| tRNA genes | 50 | 50 | 47 |
| rRNA genes (5S-16S-23S) | 10 (3 operons) | 10 (3 operons) | 6 (2 operons) |
| Protein-coding genes | 3,470 | 3,528 | 3,232 |
| Genes assigned to COGs (%) | 1,813 (52.2%) | 1,832 (51.9%) | 1,805 (55.8%) |
| Average gene length | 850.1 bp | 856.3 bp | 891.1 bp |
| Max gene length | 5,256 bp | 5,256 bp | 9,261 bp |
| ATG initiation codon proteins | 2,707 | 2,721 | 2,573 |
| GTG initiation codon proteins | 674 | 707 | 594 |
| TTG initiation codon proteins | 89 | 100 | 65 |
| CRISPR regions | 2 | - | 2 |
| **Plasmid 1** size (G+C) | 101,047 bp (52.7%) | 235,493 bp (56.9%) | - |
| DNA coding region (%) | 84,570 bp (83.7%) | 194,583 bp (82.6%) | - |
| Protein-coding genes | 84 | 209 | - |
| Genes assigned to COGs (%) | 13 (15.5%) | 93 (44.5%) | - |
| Average gene length | 1,006.8 bp | 931 bp | - |
| Max gene length | 4,350 bp | 7,983 bp | - |
| ATG initiation codon proteins | 65 | 154 | - |
| GTG initiation codon proteins | 12 | 46 | - |
| TTG initiation codon proteins | 7 | 9 | - |
| **Plasmid 2** size (G+C) | 166,472 bp (56.7%) | - | - |
| DNA coding region (%) | 140,643 bp (84.5%) | - | - |
| Protein-coding genes | 154 | - | - |
| Genes assigned to COGs (%) | 70 (45.5%) | - | - |
| Average gene length | 913.3 bp | - | - |
| Max gene length | 11,121 bp | - | - |
| ATG initiation codon proteins | 115 | - | - |
| GTG initiation codon proteins | 36 | - | - |
| TTG initiation codon proteins | 3 | - | - |

**Table S3** NCBI blastx results for CRISPR associated proteins in AArc1^T^.

| *locus_tag* | *Gene* | *Blastx best hit (nr)* | *Max score* | *Identity* | *E value* | *Accession* |
| --- | --- | --- | --- | --- | --- | --- |
|  |  |  |  |  |  |  |
| AArc1_1161 | Csa3 | *Halococcus thailandensis* | 232 | 50% | 8e-74 | WP_007736989.1 |
| AArc1_1160 | Cas6 | *Halococcus thailandensis* | 417 | 68% | 5e-145 | WP_007736987.1 |
| AArc1_1159 | Cas10d | *Natrialba hulunbeirensis* | 1229 | 79% | 0 | WP_006651363.1 |
| AArc1_1158 | Cas7 | *Natrialba hulunbeirensis* | 516 | 86% | 0 | WP_006651365.1 |
| AArc1_1157 | Cas5 | *Natrialba hulunbeirensis* | 414 | 85% | 2e-145 | WP_006651366.1 |
| AArc1_1156 | Cas3 | *Natrialba hulunbeirensis* | 929 | 72% | 0 | WP_006651367.1 |
| AArc1_1155 | Cas4 | *Natrialba hulunbeirensis* | 358 | 83% | 1e-124 | WP_006651368.1 |
| AArc1_1154 | Cas1 | *Natrialba hulunbeirensis* | 579 | 82% | 0 | WP_049912347.1 |
| AArc1_1153 | Cas2 | *Hrr. lacusprofundi* ATCC 49239 | 158 | 79% | 9e-49 | ACM59086.1 |
|  |  |  |  |  |  |  |

**Table S4** NCBI blastx results for CRISPR associated proteins in AArc-Sl.

| *locus_tag* | *Gene* | *Blastx best hit (nr)* | *Max score* | *Identity* | *E value* | *Accession* |
| --- | --- | --- | --- | --- | --- | --- |
|  |  |  |  |  |  |  |
| AArcSl_0547 | Csa3 | *Halopiger goleimassiliensis* | 199 | 47% | 7e-61 | WP_049928776.1 |
| AArcSl_0548 | Cas6 | *Halopiger goleimassiliensis* | 366 | 62% | 1e-124 | WP_049928777.1 |
| AArcSl_0549 | Cas10d | *Natrialba asiatica* | 775 | 51% | 0 | WP_049904832.1 |
| AArcSl_0550 | Cas10d | *Haloferax larsenii* | 158 | 58% | 6e-47 | WP_074796626.1 |
| AArcSl_0551 | Cas7 | *Haloferax larsenii* | 458 | 70% | 9e-160 | WP_074796628.1 |
| AArcSl_0552 | Cas5 | *Natrialba asiatica* | 286 | 59% | 1e-94 | WP_006111344.1 |
| AArcSl_0553 | Cas3 | *Halarchaeum acidiphilum* | 562 | 47% | 0 | WP_020221135.1 |
| AArcSl_0554 | Cas4 | *Haloferax larsenii* | 268 | 72% | 2e-88 | WP_074796634.1 |
| AArcSl_0555 | Cas1 | *Natronomonas pharaonis* | 562 | 86% | 0 | WP_011323349.1 |
| AArcSl_0556 | Cas2 | *Halococcus thailandensis* | 156 | 81% | 6e-48 | WP_007736979.1 |
|  |  |  |  |  |  |  |

**Table S5.** Carbon and energy metabolism of natronoarchaeal strains AArc1, Aarc-Mg and Aarc-Sl. *Separated Excel file*

**Supplementary Data 1** Proteome of the AArc1 chromosome, with nemPAI values of proteins detected in corresponding aerobic- (green), DMSO- (red) and sulfur-respiring (blue) cells. *Separated Excel file*

**SUPPLEMENTARY DISCUSSION**

*CRISPR-Cas system analysis*

A complete CRISPR system (Clustered Regularly Interspaced Short Palindromic Repeats) with associate Cas proteins (Deveau et al., 2010) was detected inside the AArc1^T^ and AArc-Sl genomes. Both share the same operon structure, with eight CRISPR associated proteins detected (Cas2, Cas1, Cas4, Cas3, Cas5, Cas7, Cas10d, and Cas6), plus a CRISPR accessory protein (DNA-binding Csa3), and two CRISPR-cassette placed at the ends (Supplementary Fig. S7, Table S3 and S4). Strain AArc1^T^ possesses 46 spacers with repeater consensus 5’- GTTTCAATCCCGTGCTGGGTTTTCTCTCCGTCGCGAC -3’ for CRISPR1-cassette, and 34 spacers with repeater consensus 5’- GTCGCAGGACACCGAAAACCCAGAACGGGATTGAAAC -3’ for CRISPR2-cassette. Strain AArc-Sl, instead, possesses 27 spacers and relative repeaters 5’- GTTTCAATCCCGTGCTGGGTTTTCTCCCTGCTGCGAC -3’) for CRISPR1, 34 spacers and 35 repeaters (5’- GTCGCAGGAGTCAGAAAACCCGAACCGGGATTGAAAC -3’) for CRISPR2. None of the spacers are in common among them. The classification for this CRISPR-Cas system was ambiguous for determining the right subtype affiliation, with a mix of I-D Cas proteins arranged in the I-B order (Makarova et al., 2011 and 2015, Koonin et al., 2017), which could be considered as a variant of the subtype I-D. The same variant was found inside other genomes of the class *Halobacteria*. In particular, *Haloarcula* sp. CBA1115 possesses also the double structure of CRISPR-cassettes described above.

**SUPPLEMENTARY METHODS**

*Genome comparisons*

Average nucleotide identity (ANI) was calculated using the BLAST (ANIb) and MUMmer (ANIm) algorithms performed by JSpecies online tool (Richter and Rosselló-Móra, 2009, http://jspecies.ribohost.com/jspeciesws/), while genome-to-genome distance analysis using digital DNA-DNA hybridization (dDDH) technique was performed by Genome-to-Genome Distance Calculator 2.1 online submission form (GGDC, Meier-Kolthoff et al., 2013, http://ggdc.dsmz.de/ggdc.php). Finally, percentage of conserved proteins (POCP) was conducted using the procedure described in Qin et al, 2014.

**REFERENCES**

Deveau H, Garneau JE, Moineau S. (2010). CRISPR/Cas system and its role in phage-bacteria interactions. *Annu Rev Microbiol* **64**: 475−493.

Makarova KS, Haft DH, Barrangou R, Brouns SJ, Charpentier E, Horvath P *et al*. (2011). Evolution and classification of the CRISPR-Cas systems. *Nat Rev Microbiol.* **9**: 467−477.

Makarova KS, Wolf YI, Alkhnbashi OS, Costa F, Shah SA, Saunders SJ *et al*. (2015). An updated evolutionary classification of CRISPR-Cas systems. *Nat Rev Microbiol* **13**: 722−736.

Koonin EV, Makarova KS, Zhang F. (2017). Diversity, classification and evolution of CRISPR-Cas systems. *Curr Opin Microbiol* **37**: 67−78.

Meier-Kolthoff JP, Auch AF, Klenk HP, Göker M. (2013). Genome sequence-based species delimitation with confidence intervals and improved distance functions. *BMC Bioinformatics* **14**: 60.

Qin QL, Xie BB, Zhang XY, Chen XL, Zhou BC, Zhou J *et al*. (2014). A proposed genus boundary for the prokaryotes based on genomic insights. *J Bacteriol* **196**: 2210−2215.

Richter M, Rosselló-Móra R. (2009). Shifting the genomic gold standard for the prokaryotic species definition. *Proc Natl Acad Sci U S A* **106**: 19126−19131.
